# Genomic and phenotypic characterization of plasmid-mediated extensively drug-resistant *Salmonella* Typhi from Lahore Pakistan carrying IncY IncQ1 and IncC replicons

**DOI:** 10.1038/s41598-026-37560-5

**Published:** 2026-03-16

**Authors:** Iqra Jamil, Asad Ur Rehman, Muhammad Fayyaz Ur Rehman, Farhan Rasheed

**Affiliations:** 1 Dr. Ikraam Ul Haq Institute of Industrial Biotechnology, Government College University Lahore, Lahore, Pakistan; 2 Institute of Chemistry, University of Sargodha, Sargodha, Pakistan; 3 Ammer Ud Din Medical College/Post Graduate Medical Institute, Lahore, Pakistan

**Keywords:** S. Typhi, Extensively drug resistant, Antimicrobial sensitivity testing, Typhoid fever, Biological techniques, Genetics, Microbiology, Medical research

## Abstract

Antimicrobial resistance and emergence of MDR and XDR *Salmonella enterica subsp. enterica* serovar Typhi is a major threat in Pakistan. Failure of treatment with conventional antibiotics leads to importance of exploring alternative treatment plan. Therefore, this study aimed to check the resistance patterns and delineate the molecular basis of resistance among clinical isolates of *S*. typhi. 384 clinical isolates of S. Typhi were processed according to standard microbiological techniques. Antimicrobial sensitivity testing was performed by using VITEK-2 compact system. DNA was extracted and Gene specific PCR was done to confirm XDR *S.* Typhi. Whole Genome Sequencing was performed and Plasmidfinder software was used for plasmids identification. Among 382 S. Typhi, males were predominant which accounted for 61.7% and 43.2% samples were from age group of less than 10 years. Carbapenems and azithromycin showed 100% sensitivity. XDR S. Typhi accounted for 38% of total isolates. Most commonly found plasmids were IncQ1, IncY and IncC. Whole genome assemblies generated using SPAdes v3.15.5 achieved a mean coverage depth of ~ 125 × , revealing the principal plasmid replicons IncQ1, IncY, IncC, and IncA/C2 associated with multidrug resistance in Salmonella Typhi. XDR Salmonella Typhi poses a significant challenge in the treatment of typhoid fever and serious threat for public health. Horizontal transfer of plasmids among Enterobacterales contributes to the emergence and maintenance of XDR S. Typhi.

**Supplementary Information:**

The online version contains supplementary material available at 10.1038/s41598-026-37560-5.

## Introduction

Typhoid fever is considered as a multisystemic disease which is caused by *Salmonella enterica*, *subsp. enterica* serotype Typhi commonly known as S. Typhi is a serious global health concern with high morbidity and mortality rate. Typhoid fever accounted for approximately 21.6 million cases and about 128,000 to 161,000 mortalities across the globe per year^1^. More than 80% cases and deaths have been reported in Asian subcontinent, that makes the situation more alarming in this region. In context of incidence rate of typhoid fever, Pakistan ranks second in Southeast Asia with almost 493.5 new reported cases per 100,000 population^2^. In some specific areas of Pakistan where typhoid is endemic, the prevalence rate of XDR typhoid has increased drastically from 7/100,000 to 15/100,000^3^. According to a report by WHO, about 10,365 cases of XDR S. Typhi have been reported by 2019. Since September 2020, a remarkable increase in extensively drug resistant isolates of *S*. Typhi have been reported in Pakistan^4^. According to the Global Burden of Disease Study 2017, an estimated 14.3 million cases of typhoid and paratyphoid fever occurred globally in 2017, resulting in ~ 136 000 deaths, with *Salmonella* Typhi accounting for ≈ 76% of all enteric-fever infections. First outbreak of extensively drug-resistant (XDR) Salmonella Typhi (H58 lineage) was identified in Hyderabad, Sindh, in late 2016, marking the emergence of a ceftriaxone-resistant strain that left only azithromycin and carbapenems effective. According to NIH Pakistan surveillance and WHO outbreak reports, the infection spread rapidly within Sindh by 2018, over 5,000 confirmed XDR cases had been recorded, mostly from Karachi (≈69%) and Hyderabad (≈27%)^5^. Between 2019 and 2021, the outbreak extended nationwide to Punjab, Khyber Pakhtunkhwa, and Balochistan, with XDR strains accounting for nearly 70% of all *S*. Typhi infections^6^. Provincial surveillance further indicated that the incidence of XDR typhoid doubled from roughly 7 to 15 per 100,000 population, highlighting its rapid spread and establishing Pakistan as the global epicenter of XDR typhoid fever.

Drastic increase in antimicrobial resistance in typhoidal salmonella isolates against antityphoidal drugs results in disease severity along with increased antibiotic treatment cost and also need for hospitalization of sever cases which eventually results in burden on healthcare. Since the emergence of antibiotic resistance against first-line drugs by *Salmonella* Typhi strains, these antimicrobial drugs fell out of favor and hence not used frequently for this illness lately. Since the emergence of multiple drug resistant isolates of *S*. Typhi which are collectively resistant to ampicillin, chloramphenicol and co-trimoxazole, ciprofloxacin becomes the drug of choice for the treatment of enteric fever^7^. The *S*. Typhi strains described as extensively drug resistant (XDR) are actually MDR strains with acquire additional resistance against fluoroquinolone and cephalosporins class, leaving only azithromycin and carbapenem as the drugs of last resort. However, emerging regional data indicate gradual azithromycin MIC elevation and sporadic resistance, suggesting that sole reliance on this agent as a last-line oral therapy may be precarious. After the first outbreak of XDR S. Typhi in Sindh, more than 17,000 XDR S. Typhi cases from this region have been reported according to a report of National Institute of Health, Pakistan^1^. In Karachi, approximately 14,360 XDR cases of typhoid fever were reported from January 2017 to June 2021^8^.

The most effective protocol for the detection of increase in antibiotic resistance is laboratory surveillance. In this era of evolving mechanisms of resistance, a continuous study of the basis of antimicrobial resistance against existing drugs accomplished by both phenotypic and genotypic analysis can help in formulating alternative therapeutic approaches and targets for the treatment of infection caused by multidrug resistant bacterial isolates. Antimicrobial resistance in bacterial isolates may be intrinsic or extrinsic which can be acquired by either mutation in genes of chromosomes or by acquisition of resistance determinants which are mediated by diverse plasmids. Selection pressure mainly leads to development of the intrinsic resistance in bacterial isolates whereas acquisition of extrinsic genes is commonly associated with horizontal gene transfer^9^.Among these plasmids, the IncY replicon often harbors extended-spectrum β-lactamase (ESBL) genes such as blaCTX-M variants; IncQ1 commonly carries *sul2, strA/B*, and *qacEΔ1* determinants conferring sulfonamide and streptomycin resistance; while IncC/IncA/C2 plasmids act as broad multidrug-resistance backbones frequently co-localizing multiple AMR genes across diverse Gram-negative pathogens^10^.

IncY plasmids typically harbor ESBL genes like blaCTX-M variants, IncQ1 often carries *sul2, strA/B*, and *qacEΔ1*, while IncC/IncA/C2 serve as broad MDR backbones, frequently co-localizing multiple resistance determinants.With the advancement of latest techniques, whole genome sequencing (WGS) has been used more extensively for understanding of emergence of resistance mechanisms in bacterial isolates and it also help to track the spread of bacterial and other microbial pathogens^9^. Results of WGS of individual XDR S. Typhi cases of from Canada, Denmark and Taiwan have shown a very close relationship with almost 100% match of nucleotide to the nearest XDR sequenced isolate from Pakistan^11^. Therefore, the present study was aimed to determine the susceptibility pattern of S. Typhi isolates towards commonly used antityphoidal drugs from the febrile individuals attending the tertiary care hospital at Lahore, Pakistan. We report the emergence of extensively drug-resistant (XDR) isolates obtained from the blood cultures obtained from these febrile patients and have delineated the genetic basis of cephalosporin resistance. We hypothesized that XDR isolates from Lahore would harbor ESBL-bearing IncY and/or IncQ1 plasmid replicons, consistent with their observed cephalosporin and fluoroquinolone resistance phenotypes.

## Materials and methods

This was a cross-sectional descriptive study conducted at Pathology department of Allama Iqbal Medical College and 3 years from March 2021 to March 2024. Ethical approval was taken from Ethical Review Board (ERB) of Allama Iqbal Medical College/ Jinnah Hospital Lahore Reference no: 47th/ERB held on 21st March 2021. All methods were carried out as per the guidelines and regulations of Ethical Review Board. An informed consent was taken from each participant of the study. Blood cultures were collected from the suspected cases of typhoid fever and were processed as per the routine diagnostic protocols. Patients who had received systemic antibiotic therapy within the preceding 14 days or those with non-typhoidal Salmonella infections were excluded. Blood samples were collected from patients presenting to the emergency department, pediatric and medical wards, and outpatient clinics of the same institution. Only the first positive blood culture per patient was included in the analysis to avoid duplicate entries. A total of 384 isolates were obtained during the study period. This sample size provided a post-hoc precision of approximately ± 4.8% for the estimated XDR prevalence of 38% at a 95% confidence level. Bacterial identification was done by using colonial morphology, gram staining, and different biochemical tests. VITEK 2 (bioMérieux, France; software version 9.02) compact automated identification and antimicrobial susceptibility testing instruments were used for the antimicrobial sensitivity testing. PCR amplification for detection of XDR/MDR‐associated loci was performed using newly designed primers that are currently submitted for patent protection at Pakistan IPO. For the target region NZ_WNTL0100.1:4836–4893, the primers used were: forward 5′-ATCGTGGAACAAAGGCCGTAT-3′ and reverse 5′-TCCACCCATGAAAAATCCGGT-3′, which produce a 603-bp amplicon.

Isolates were defined as multidrug-resistant (MDR) when resistant to all three first-line antityphoidal drugs ampicillin, chloramphenicol, and co-trimoxazole and as extensively drug-resistant (XDR) when additionally resistant to fluoroquinolones and third-generation cephalosporins while remaining susceptible to azithromycin and carbapenems, in alignment with the definitions used in Table 1. For quality assurance, minimum inhibitory concentrations (MICs) of key antimicrobial agents—ceftriaxone, ciprofloxacin, and azithromycin—were confirmed by E-test (bioMérieux) using Mueller–Hinton agar, interpreted according to CLSI 2023 breakpoints.DNA extraction was performed using Promega Wizard Kit as instructed by the manufacturer. The samples were quantified using NanoDrop with respect to their volume and run on the gel. Agarose Gel Electrophoresis was performed using 1%Agarose Gel at a voltage of 150 V for 40 min.

**Table 1:** Antimicrobial sensitivity pattern of XDR, MDR and Wild isolates of *Salmonella* Typhi.

Antibiotic class	Antibiotic	Sensitivity pattern XDR (n = 144)		Sensitivity pattern MDR (n = 136)		Sensitivity pattern WILD (n = 104)	
		Sensitive (S)	Resistant (R)	Sensitive (S)	Resistant (R)	Sensitive (S)	Resistant (R)
Aminopenicillins (n = 384)	Ampicillin (AMP)	0 (0%)	144 (100%)	42 (30.9%)	94 (69.1%)	84 (80.8%)	20 (19.2%)
Anti-Folate (n = 384)	Co-trimoxazole (SXT)	0 (0%)	144 (100%)	48 (35.3%)	88 (64.7%)	104 (100%)	0 (0%)
Carbapenems (n = 384)	Imipenem (IPM)	144 (100%)	0 (0%)	136 (100%)	0 (0%)	104 (100%)	0 (0%)
	Meropenem (MEM)	144 (100%)	0 (0%)	136 (100%)	0 (0%)	104 (100%)	0 (0%)
Cephalosporins (n = 384)	Ceftriaxone (CTX)	0 (0%)	144 (100%)	136 (100%)	0 (0%)	104 (100%)	0 (0%)
Fluoroquinolones (n = 384)	Ciprofloxacin (CIP)	0 (0%)	144 (100%)	30 (22.1%)	106 (77.9%)	15 (14.4%)	89 (85.6%)
Macrolides (n = 384)	Azithromycin (AZM)	144 (100%)	0 (0%)	136 (100%)	0 (0%)	104 (100%)	0 (0%)
Pristinamycin (n = 384)	Chloramphenicol (C)	0 (0%)	144 (100%)	46 (33.8%)	90 (66.2%)	98 (94.2%)	6 (5.8%)

### Whole genome sequencing

BGI’s DNBSEQ™ platform was used for the whole genome sequencing of XDR S. Typhi. This platform uses a distinctive approach to short-read library preparation, integrating advanced technologies to enhance sequencing accuracy and efficiency. This further involves the creation of DNA Nanoballs (DNBs) through linear Rolling Circle Replication (RCR), which amplifies circularized DNA molecules without introducing the biases commonly associated with PCR amplification. The DNBSEQ short-read library preparation and sequencing protocol involves the following steps: (a) DNA fragmentation, (b) Size selection, (c) End repair and “A” tailing, (d) Adapter ligation, (e) Product size selection, (f) PCR reaction and (g) Library QC. A total of five genomes (52E, 54E, 55E, 56E, and 58E) representing wild-type and multidrug-resistant (MDR) *S*. Typhi isolates were sequenced using the BGI DNBSEQ™ platform. The assembled genomes were of overall good quality, generated using SPAdes v3.15.5 with 500 bp minimum contig cutoff and mean coverage depth of ~ 125 × . For isolate 52E, the assembly comprised 392 contigs, total length 4,927,938 bp, GC content 51.98%, and N50 = 174,576 bp. Isolate 54E produced 78 contigs (total 4,812,103 bp, GC 52.03%), while 55E showed a lower-quality assembly (10,749 contigs, 16,892,477 bp, GC 44.53%). The 56E genome comprised 906 contigs (9,602,749 bp, GC 47.00%), and 58E showed 268 contigs (4,868,150 bp, GC 51.99%). Mean Q30 scores exceeded 92% across all libraries. Plasmid replicon screening was performed using PlasmidFinder v2.1 (database updated Feb 2024) with 95% identity and 80% coverage thresholds, identifying principal plasmids IncY, IncQ1, and IncA/C2, consistent with MDR and XDR plasmid profiles described in literature. Outputs include a tabular report listing plasmid names, their corresponding replicon families, percent identity, and query coverage. These outputs provide insights into the diversity and abundance of plasmids in each genome.

AMR gene annotation was performed using AMRFinder, which identified key resistance genes in the sequenced genomes. This annotation helps to strengthen the genomic narrative by associating the resistance profiles of the isolates with known genetic determinants of antimicrobial resistance.

## Results

A total of 384 isolates were collected over the study period, with 61.7% (n = 237) of the samples being male and 38.3% (n = 147) being female. The age distribution showed that the under 10 years age group was the most affected, accounting for 43.2% (n = 166) of the total samples, followed by 24.2% (n = 93) from the 10 to 20 years age group, and 19.6% (n = 75) from the 20 to 30 years age group. Only 2.3% (n = 9) of the samples were collected from individuals over 50 years of age. A supplementary histogram illustrating the age distribution of the isolates is shown below. For the antimicrobial resistance (AMR) profiles, the isolates were categorized as multidrug resistant (MDR) or extensively drug-resistant (XDR) based on their resistance to key antibiotics. A total of 38% (n = 144) isolates were XDR, 35% (n = 136) were MDR, and 27% (n = 104) were wild isolates. Comparative statistics were performed to assess the association between XDR frequency and age group/sex. A chi-squared test (χ^2^) was performed on XDR frequency by sex and age group, which revealed a statistically significant difference (χ^2^ = 11.60, *p* = 0.0089), indicating that sex and age group are associated with XDR prevalence. Resistance pattern of S. Typhi isolates shown in Table 1, the total number of isolates in the study was 384, with XDR, MDR, and wild isolates representing different resistance profiles.* P*-values were calculated to compare the resistance patterns between XDR, MDR, and wild isolates for each antibiotic. All comparisons showed statistically significant differences (*p* < 0.05), indicating substantial variation in resistance patterns across these groups.

Minimum inhibitory concentrations (MICs) of the tested drugs were determined for the confirmed isolates of S. Typhi: Table 2.

**Table 2:** MICs of XDR, MDR and Wild isolates of S. Typhi.

Antibiotic	MIC range (µg/mL) of XDR Isolates	MIC range (µg/mL) of MDR Isolates	MIC range (µg/mL) of Wild Isolates	MIC50 (µg/mL)	MIC90 (µg/mL)
Ampicillin	≥ 32	≤ 4 to ≥ 32	≤ 2 to ≥ 32	32	32
Co-trimoxazole	20 to ≥ 320	≤ 20 to ≥ 320	≤ 2	320	320
Imipenem	≤ 0.25	≤ 0.25	≤ 0.25	0.25	0.25
Meropenem	≤ 0.25	≤ 0.25	≤ 0.25	0.25	0.25
Ceftriaxone	≥ 64	≤ 1	≤ 1	64	64
Ciprofloxacin	1 to ≥ 4	≤ 0.25 to ≥ 4	≤ 0.25 to ≥ 4	4	4

DNA extraction was performed using Promega DNA extraction kit, and gene specific PCR was run to confirm the extensively drug resistance pattern on molecular basis. Supplementary Fig. 1.

In this study, we performed whole-genome sequencing for XDR isolates. The basic assembly quality control (QC) metrics are shown in Table 3 and were computed from the FASTA files as follows:

**Table 3:** WGS assembly quality control metrics for XDR isolates.

Isolate	Contigs	Total length (bp)	N50 (bp)	Max Contig (bp)	GC content (%)
LHR-XDR-01 (52E)	392	4,927,938	149,295	488,885	51.98
LHR-XDR-02 (54E)	372	4,894,017	149,295	428,629	51.98
LHR-XDR-03 (54E)	78	4,812,103			52.03%
LHR-XDR-03 (56E)	346	4,873,673	149,295	392,779	51.95
LHR-XDR-04 (58E)	268	4,868,150	173,739	488,885	51.99

After confirmation of XDR, whole genome sequencing was performed for the identification of plasmids which confer resistance to different antimicrobial drugs. Plasmid Identification was performed using PlasmidFinder software. In comparing the plasmid profiles across the XDR genomes, several patterns and unique distinctions emerge. The plasmid IncY is consistently identified in all five XDR genomes (52E, 54E, 55E, 56E, and 58E), showing 98.82% identity across datasets, suggesting its essential role in conferring antibiotic resistance in these strains. Similarly, the plasmid IncQ1, with 100% identity, is shared by genomes 52E, 54E, 55E, and 58E, but absent in 56E, highlighting its varying presence among closely related strains. Genomes 55E and 56E exhibit additional plasmids that distinguish them from other XDR genomes. Notably, 55E includes plasmids Col3M, ColpVC, and IncC, which are absent in other XDR strains, signifying a broader repertoire of plasmid-associated resistance genes. On the other hand, 56E shares Col3M and ColpVC with 55E but lacks the IncQ1 plasmid, underlining the variation in plasmid content even within the XDR category Table 4.

**Table 4:** Plasmids sequencing isolated from XDR S. Typhi.

Sample ID	Plasmid	%Identity	HSP length/total length	Start	End	Accession
52E	IncQ1	100	529/796	3940	4468	M28829
	IncY	98.82	765/765	3873	3109	K02380
54E	IncQ1	100	529/796	1114	1642	M28829.1
	IncY	98.82	765/765	3109	3873	K02380
55E	Col3M	98.09	157/157	482	638	JX514065
	ColpVC	98.45	193/193	1209	1401	JX133088
	IncC	100	417/417	15,451	15,867	JN157804
	IncQ1	100	529/796	599	71	M28829
	IncY	98.82	765/765	3873	3109	K02380
56E	Col3M	98.09	157 / 157	3448	3604	JX514065
	ColpVC	98.45	193 / 193	108	300	JX133088
	IncA/C2	100	417 / 417	54,940	55,356	JN157804
58E	IncQ1	100	529 / 796	123	651	M28829
	IncY	98.82	765 / 765	2767	3531	K02380

The XDR genomes’ distinct resistance profiles, especially in 55E, underscore their evolutionary adaptation to diverse antimicrobial pressures. These findings emphasize the complexity of resistance mechanisms, wherein the presence of resistance genes does not always translate into an observable phenotype, highlighting the importance of studying gene expression and regulatory networks.

AMR genes were identified for each isolate using AMRFinderPlus and ResFinder. The plasmid replicons were determined using PlasmidFinder. The results showed that XDR isolates (LHR-XDR-01 to LHR-XDR-04) harbored various plasmid replicons associated with specific AMR genes. For instance, LHR-XDR-01 (52E) and LHR-XDR-02 (54E) both contained IncY and IncQ1 plasmids and exhibited an ESBL-producing and multidrug-resistant phenotype, with blaCTX-M-15 being a key resistance gene. LHR-XDR-03 (56E) carried IncA/C2, Col3M, and ColpVC plasmids, associated with AmpC-producing and multidrug-resistant phenotypes. Lastly, LHR-XDR-04 (58E), like the other two, contained IncY and IncQ1 plasmids, with blaCTX-M-15 as the prominent ESBL gene. For a full list of AMR genes and their corresponding plasmid replicons across all isolates, please refer to Supplementary Table S1.

## Discussion

The results of this study encompass extensive characterization of Salmonella Typhi isolates over two years from a tertiary care hospital in Lahore. The Lahore dataset aligns with national trends, showing similar IncY and IncQ1 plasmids in Sindh. However, Peshawar exhibits a higher prevalence of IncA/C2 and Col plasmids, highlighting regional differences in AMR gene profiles. This underscores the importance of continued surveillance across regions to understand the spread of multidrug-resistant and extensively drug-resistant S. Typhi. High fever along with systemic signs and symptoms is an indication for urgent treatment with antibiotics. Drug-resistant strains of typhoid present an enormous challenge for healthcare systems in Pakistan and around the world. The emergence of an extensively drug-resistant strain of Salmonella Typhi in Pakistan in 2016 has resulted in a number of local outbreaks and an increasing number of travel-associated cases all over the world^12^. The results of the study provide detailed information about the demographics, resistance to antimicrobial agents, and genomic characterization of these isolates, thus providing valid information on the epidemiology and mechanisms of resistance constituting *S*. Typhi. The observed male predominance (61.7%) and the high proportion of cases in children under 10 years of age (43.2%) are consistent with patterns reported in previous studies. These demographic trends are likely influenced by sociobehavioral determinants, including exposure risks (e.g., water and foodborne contamination, hygiene practices) and care-seeking behaviors. Young children, in particular, may be more vulnerable due to underdeveloped immunity and increased exposure to pathogens in household environments. Studies have shown that males are often at higher risk due to behavioral factors and greater outdoor activity, which can increase exposure to environmental contaminants. Furthermore, age-specific healthcare access and care-seeking patterns are critical in understanding the higher rates of infection in younger populations^13–15^. This observation coincides with the previous works that report typhoid fever usually attacks younger individuals due to their not-so-well developed immunity and exposure to the organism through contaminated food and water^14^. These findings were in agreement with other studies conducted in Pakistan and international studies^15–18^. The outcome of antimicrobials susceptibility tests displayed alarming resistance patterns found in *S*. Typhi isolates. In detail, the isolates were resistant to 67.2% of ampicillin, 60.4% of co-trimoxazole, 37.5% of ceftriaxone, and 88.3% of ciprofloxacin, but 100% sensitive to carbapenems (imipenem and meropenem) and azithromycin. It is worthwhile to point out that some studies have shown that ever-increasing resistance to azithromycin might influence treatment failure. The high resistance rates shown by fluoroquinolones and third-generation cephalosporins are especially worrying because these two are nearly always the first-line treatment for typhoid fever. Yousaf et al. shows same resistance pattern in salmonella isolates from the Patients in Pakistan^19^. The observed resistance patterns in *S*. Typhi isolates in Lahore are consistent with findings from other regions in Pakistan. Studies from Karachi and Peshawar have also reported high resistance to ampicillin, co-trimoxazole, ciprofloxacin, and ceftriaxone, though the proportions vary across regions^18,20,21^. It is in fact more resistant than the earlier studies from Karachi^21,22^ but correlates with findings from yet more recent research in the same region under mention^23,24^. The global situation is also alarming as the resistance to first-line drugs varies—showing that Pakistan is alarming concerning the data available to support this claim^16–18,25^. Though some researchers presented more significant susceptibility data related to ciprofloxacin^16,18,20^ local research within tertiary care hospitals across Pakistan continues to reflect some of the similar susceptibilities^21–24^. The already existing free ciprofloxacin availability has made it easy to acquire for the treatment of various infections. The azithromycin susceptibility in our study was also 100%, which is consistent with Karachi isolates; however, emerging azithromycin resistance has been reported in some regions of Pakistan (Khan et al., 2019), signaling the need for continuous monitoring. Primarily recorded to emerge in November 2016, resistance to ceftriaxone occurred in a major outbreak in Hyderabad, Pakistan, which turned into a nationwide spread^20,23,25–28^. Lower resistance rates toward ceftriaxone have also been reported by some local studies, which go in line with our findings^15,20^. In contrast to this, other studies conducted in Pakistan had reported an increased trend of ceftriaxone resistance^23^. Also, international studies have shown ceftriaxone resistance among people who traveled to and from Pakistan^29^. All isolates from our study were sensitive to azithromycin; however, earlier studies implicated that the easy availability of this drug without prescriptions in Pakistan and its use widely prophylactically during the COVID-19 pandemic have been contributing to resistance and decrease in efficacy in Pakistan^15,20,23,26^ as well as from global perspectives^17,18^. Given the current trends in resistance, further regional studies are needed to track possible emerging patterns of local antimicrobial susceptibility. This will aid in developing evidence-based guidelines for empirical treatment of typhoid cases. The resistance types that the isolates were classified into include: extensively drug resistant (XDR), multidrug resistant (MDR) and wild type strains. Of the total isolates, XDR accounted for about 37.5%- that is, it resisted five or more classes of related antibiotics including third generation cephalosporins. MDR comprised 35.4% and, therefore, resisted three classes of antibiotics. Several reports showed the increasing burden of multi-drug-resistant *Salmonella* Typhi in various parts of the world like China, India, Indonesia, Pakistan, and Vietnam^30^. Some other studies have reported the MDR stains of salmonella from Nepal^30^. The H58 lineage of S. Typhi dominates the global spread of drug-resistant strains, with typical SNP distances showing close phylogenetic relatedness to isolates from Southeast Asia and Africa, indicating shared transmission dynamics. This emphasizes the need for global surveillance to monitor the cross-border spread of resistant *S*. Typhi. Wild type strains (27.1%) were mostly susceptible to most of the antibiotics tested. The predominance of XDR and MDR isolates emphasizes that typhoid fever became difficult to manage as well as gives the rationale for continuous surveillance. Further insights into the resistance mechanism were revealed by genomic analysis carried out with the selected isolates. The PCR-based discrimination revealed resistance gene cassettes in the XDR strains but not in the wild-type strain. Also identified resistance plasmids through whole genome sequencing of the 17 isolates were IncA/C2, IncFIB(pHCM2), IncQ1, and IncY. The identification of plasmid-built resistance genes such as blaCMY-6, qnr, and aadA2 showed the contribution of horizontal gene transfer in the dissemination of antimicrobial resistance. These results are supported by the findings of other studies^31,32^. A study from Peshawar reported partial sequence of IncQ1 plasmid was characterized for WGS data of samples. Furthermore, ceftriaxone-resistant IncY plasmid was identified in one strain of XDR10 from Peshawar^32^. Reports of IncY plasmids in XDR isolates of Salmonella Typhi from the Sindh and Punjab regions of Pakistan have been documented^1^. HGT events have been reported in *Acinetobacter spp.*, *Pseudomonas spp*., and *Salmonella spp*., where innate resistance is mainly plasmid-mediated. Genomic analysis of XDR S. Typhi isolates from Peshawar confirmed the presence of the blaCTX-M-15 gene in all samples. Prior reports of this plasmid have been limited to a handful of XDR S. Typhi isolates from Punjab province, Pakistan^33^. Through comparative analysis, the study suggests that the XDR strains were more heavily loaded with plasmids and resistance determinants in comparison to the wild-types. The weight of evidence adduced in this study shows that S. Typhi isolates have a high level of antimicrobial resistance, with a considerable proportion being classified as XDR or MDR. These resistance patterns and genomic evidence form a vantage point from which antimicrobial stewardship shall consider alternative treatment strategies. Given the high resistance to third-generation cephalosporins (e.g., ceftriaxone) observed in this study, we recommend avoiding their use as first-line therapy in Lahore and considering alternative agents. Azithromycin may be a viable option, but susceptibility testing should be conducted to ensure effectiveness, as emerging resistance is a concern. For severe cases, carbapenems should be preserved as a last-resort therapy due to their effectiveness against *S*. Typhi in our dataset. This approach will help reduce the overuse of broad-spectrum antibiotics and contribute to controlling the spread of resistant strains in the region. This study has several limitations. First, the sampling was restricted to a single city (Lahore), which may limit the generalizability of the findings to other regions of Pakistan or beyond. Second, short-read only assemblies were used, which may have limited plasmid resolution and may not fully capture all plasmid-related resistance determinants. Third, functional assays (e.g., conjugation studies and transfer frequency assays) were not performed, which would have provided more insight into the horizontal gene transfer of resistance. Additionally, we did not perform a standardized phylogenetic lineage assignment, which could have more clearly positioned our isolates within global S. Typhi transmission dynamics. Lastly, expression-level confirmation (e.g., ESBL activity assays) was not included, which would have validated the presence of resistance genes at the functional level. Future evaluation may be required for novel therapies and possible vaccination efforts to curtail the dissemination of resistant S. Typhi strains. Future studies should explore long-read hybrid assemblies to improve plasmid resolution and conduct conjugation experiments to assess gene transferability. Additionally, metagenomic analysis of environmental reservoirs and time-series surveillance to monitor azithromycin resistance progression are essential for controlling the spread of resistant strains.

## Conclusion

This study underscores the serious threat of XDR Salmonella Typhi which poses a significant challenge in the treatment of typhoid fever as the strains of S. Typhi become resistant both 1st and 2nd line antimicrobial drugs. High level molecular studies revealed that presence of IncQ1, IncC and IncY plasmids among the isolates of XDR S. Typhi collected from Lahore region. Moreover, validation of inheritance of identified plasmids in clinical isolates of S. Typhi play an important role in the emergence of XDR typhoid in the future.

## Supplementary Information

Below is the link to the electronic supplementary material.

Supplementary Material 1

Supplementary Material 2

Supplementary Material 3

Supplementary Material 4

Supplementary Material 5

## Data Availability

Plasmid sequence data are publicly available in Science Data Bank under CSTR accession 31253.11.sciencedb.35275 (https://cstr.cn/31253.11.sciencedb.35275; Science Data Bank short link: https://www.scidb.cn/en/s/fAvqYv).
