# Supplementary material for: Genomic and phenotypic characterization of plasmid-mediated extensively drug-resistant Salmonella Typhi from Lahore Pakistan carrying IncY IncQ1 and IncC replicons
Source: Sci Rep. 2026 Mar 16;16:13606. doi: 10.1038/s41598-026-37560-5 (PMC13121732; doi:10.1038/s41598-026-37560-5)
Supplement: Supplementary file 2 — Supplementary Material 2 [file 41598_2026_37560_MOESM2_ESM.docx]

**S1 Table:** Antibiotic resistance gene in 52E genome

| **Gene** | **Predicted Phenotype** | **CGE Predicted Phenotype** | **%Identity** | **Start** | **End** | **Accession** | **Sequence** |
| --- | --- | --- | --- | --- | --- | --- | --- |
| aph(3'')-Ib | streptomycin | Streptomycin | 99.88 | 1725 | 922 | AF321551 | TTGAATCGAACTAATATTTTTTTGGGTGAATCGCATTCTGACTGGTTGCCTGTCAGAGGCGGAGAATCTGGTGATTTTGTTTTTCGACGTGGTGACGGGCATGCCTTCGCGAAAATCGCACCTGCTTCCCGCCGCGGTGAGCTCGCTGGAGAGCGTGACCGCCTCATTTGGCTCAAAGGTCGAGGTGTGGCTTGCCCCGAGGTCATCAACTGGCAGGAGGAACAGGAGGGTGCATGCTTGGTGATAACGGCAATTCCGGGAGTACCGGCGGCTGATCTGTCTGGAGCGGATTTGCTCAAAGCGTGGCCGTCAATGGGGCAGCAACTTGGCGCTGTTCACAGCCTATCGGTTGATCAATGTCCGTTTGAGCGCAGGCTGTCGCGAATGTTCGGACGCGCCGTTGATGTGGTGTCCCGCAATGCCGTCAATCCCGACTTCTTACCGGACGAGGACAAGAGTACGCCGCAGCTCGATCTTTTGGCTCGTGTCGAACGAGAGCTACCGGTGCGGCTCGACCAAGAGCGCACCGATATGGTTGTTTGCCATGGTGATCCCTGCATGCCGAACTTCATGGTGGACCCTAAAACTCTTCAATGCACGGGTCTGATCGACCTTGGGCGGCTCGGAACAGCAGATCGCTATGCCGATTTGGCACTCATGATTGCTAACGCCGAAGAGAACTGGGCAGCGCCAGATGAAGCAGAGCGCGCCTTCGCTGTCCTATTCAATGTATTGGGGATCGAAGCCCCCGACCGCGAACGCCTTGCCTTCTATCTGCGATTGGACCCTCTGACTTGGGGTTGA |
| aph(6)-Id | kanamycin | Streptomycin | 100 | 922 | 86 | M28829 | ATGTTCATGCCGCCTGTTTTTCCTGCTCATTGGCACGTTTCGCAACCTGTTCTCATTGCGGACACCTTTTCCAGCCTCGTTTGGAAAGTTTCATTGCCAGACGGGACTCCTGCAATCGTCAAGGGATTGAAACCTATAGAAGACATTGCTGATGAACTGCGCGGGGCCGACTATCTGGTATGGCGCAATGGGAGGGGAGCAGTCCGGTTGCTCGGTCGTGAGAACAATCTGATGTTGCTCGAATATGCCGGGGAGCGAATGCTCTCTCACATCGTTGCCGAGCACGGCGACTACCAGGCGACCGAAATTGCAGCGGAACTAATGGCGAAGCTGTATGCCGCATCTGAGGAACCCCTGCCTTCTGCCCTTCTCCCGATCCGGGATCGCTTTGCAGCTTTGTTTCAGCGGGCGCGCGATGATCAAAACGCAGGTTGTCAAACTGACTACGTCCACGCGGCGATTATAGCCGATCAAATGATGAGCAATGCCTCGGAACTGCGTGGGCTACATGGCGATCTGCATCATGAAAACATCATGTTCTCCAGTCGCGGCTGGCTGGTGATAGATCCCGTCGGTCTGGTCGGTGAAGTGGGCTTTGGCGCCGCCAATATGTTCTACGATCCGGCTGACAGAGACGACCTTTGTCTCGATCCTAGACGCATTGCACAGATGGCGGACGCATTCTCTCGTGCGCTGGACGTCGATCCGCGTCGCCTGCTCGACCAGGCGTACGCTTATGGGTGCCTTTCCGCAGCTTGGAACGCGGATGGAGAAGAGGAGCAACGCGATCTAGCTATCGCGGCCGCGATCAAGCAGGTGCGACAGACGTCATACTAG |
| blaCTX-M-15 | ampicillin, ceftriaxone | Amoxicillin, Ampicillin, Aztreonam, Cefepime, Cefotaxime, Ceftazidime, Ceftriaxone, Piperacillin, Ticarcillin | 100 | 15149 | 14274 | AY044436 | ATGGTTAAAAAATCACTGCGCCAGTTCACGCTGATGGCGACGGCAACCGTCACGCTGTTGTTAGGAAGTGTGCCGCTGTATGCGCAAACGGCGGACGTACAGCAAAAACTTGCCGAATTAGAGCGGCAGTCGGGAGGCAGACTGGGTGTGGCATTGATTAACACAGCAGATAATTCGCAAATACTTTATCGTGCTGATGAGCGCTTTGCGATGTGCAGCACCAGTAAAGTGATGGCCGCGGCCGCGGTGCTGAAGAAAAGTGAAAGCGAACCGAATCTGTTAAATCAGCGAGTTGAGATCAAAAAATCTGACCTTGTTAACTATAATCCGATTGCGGAAAAGCACGTCAATGGGACGATGTCACTGGCTGAGCTTAGCGCGGCCGCGCTACAGTACAGCGATAACGTGGCGATGAATAAGCTGATTGCTCACGTTGGCGGCCCGGCTAGCGTCACCGCGTTCGCCCGACAGCTGGGAGACGAAACGTTCCGTCTCGACCGTACCGAGCCGACGTTAAACACCGCCATTCCGGGCGATCCGCGTGATACCACTTCACCTCGGGCAATGGCGCAAACTCTGCGGAATCTGACGCTGGGTAAAGCATTGGGCGACAGCCAACGGGCGCAGCTGGTGACATGGATGAAAGGCAATACCACCGGTGCAGCGAGCATTCAGGCTGGACTGCCTGCTTCCTGGGTTGTGGGGGATAAAACCGGCAGCGGTGGCTATGGCACCACCAACGATATCGCGGTGATCTGGCCAAAAGATCGTGCGCCGCTGATTCTGGTCACTTACTTCACCCAGCCTCAACCTAAGGCAGAAAGCCGTCGCGATGTATTAGCGTCGGCGGCTAAAATCGTCACCGACGGTTTGTAA |
| blaTEM-1B | ampicillin | Amoxicillin, Ampicillin, Cephalothin, Piperacillin, Ticarcillin | 100 | 437 | 1297 | AY458016 | ATGAGTATTCAACATTTTCGTGTCGCCCTTATTCCCTTTTTTGCGGCATTTTGCCTTCCTGTTTTTGCTCACCCAGAAACGCTGGTGAAAGTAAAAGATGCTGAAGATCAGTTGGGTGCACGAGTGGGTTACATCGAACTGGATCTCAACAGCGGTAAGATCCTTGAGAGTTTTCGCCCCGAAGAACGTTTTCCAATGATGAGCACTTTTAAAGTTCTGCTATGTGGTGCGGTATTATCCCGTGTTGACGCCGGGCAAGAGCAACTCGGTCGCCGCATACACTATTCTCAGAATGACTTGGTTGAGTACTCACCAGTCACAGAAAAGCATCTTACGGATGGCATGACAGTAAGAGAATTATGCAGTGCTGCCATAACCATGAGTGATAACACTGCTGCCAACTTACTTCTGACAACGATCGGAGGACCGAAGGAGCTAACCGCTTTTTTGCACAACATGGGGGATCATGTAACTCGCCTTGATCGTTGGGAACCGGAGCTGAATGAAGCCATACCAAACGACGAGCGTGACACCACGATGCCTGCAGCAATGGCAACAACGTTGCGCAAACTATTAACTGGCGAACTACTTACTCTAGCTTCCCGGCAACAATTAATAGACTGGATGGAGGCGGATAAAGTTGCAGGACCACTTCTGCGCTCGGCCCTTCCGGCTGGCTGGTTTATTGCTGATAAATCTGGAGCCGGTGAGCGTGGGTCTCGCGGTATCATTGCAGCACTGGGGCCAGATGGTAAGCCCTCCCGTATCGTAGTTATCTACACGACGGGGAGTCAGGCAACTATGGATGAACGAAATAGACAGATCGCTGAGATAGGTGCCTCACTGATTAAGCATTGGTAA |
| catA1 | Chloramphenicol | Chloramphenicol | 99.85 | 8824 | 8165 | V00622 | ATGGAGAAAAAAATCACTGGATATACCACCGTTGATATATCCCAATGGCATCGTAAAGAACATTTTGAGGCATTTCAGTCAGTTGCTCAATGTACCTATAACCAGACCGTTCAGCTGGATATTACGGCCTTTTTAAAGACCGTAAAGAAAAATAAGCACAAGTTTTATCCGGCCTTTATTCACATTCTTGCCCGCCTGATGAATGCTCATCCGGAATTCCGTATGGCAATGAAAGACGGTGAGCTGGTGATATGGGATAGTGTTCACCCTTGTTACACCGTTTTCCATGAGCAAACTGAAACGTTTTCATCGCTCTGGAGTGAATACCACGACGATTTCCGGCAGTTTCTACACATATATTCGCAAGATGTGGCGTGTTACGGTGAAAACCTGGCCTATTTCCCTAAAGGGTTTATTGAGAATATGTTTTTCGTCTCAGCCAATCCCTGGGTGAGTTTCACCAGTTTTGATTTAAACGTGGCCAATATGGACAACTTCTTCGCCCCCGTTTTCACCATGGGCAAATATTATACGCAAGGCGACAAGGTGCTGATGCCGCTGGCGATTCAGGTTCATCATGCCGTTTGTGATGGCTTCCATGTCGGCAGAATGCTTAATGAATTACAACAGTACTGCGATGAGTGGCAGGGCGGGGCGTAA |
| dfrA7 | trimethoprim | Trimethoprim | 100 | 2141 | 1668 | AB161450 | TTGAAAATTTCATTGATTTCTGCAACGTCAGAAAATGGCGTAATCGGTAATGGCCCTGATATCCCATGGTCAGCAAAAGGTGAGCAGTTACTCTTTAAAGCGCTCACATATAATCAGTGGCTCCTTGTTGGAAGGAAAACATTTGACTCTATGGGTGTTCTTCCAAATCGAAAATATGCAGTAGTGTCGAGGAAAGGAATTTCAAGCTCAAATGAAAATGTATTAGTCTTTCCTTCAATAGAAATCGCTTTGCAAGAACTATCGAAAATTACAGATCATTTATATGTCTCTGGTGGCGGTCAAATCTACAATAGTCTTATTGAAAAAGCAGATATAATTCATTTGTCTACTGTTCACGTTGAGGTTGAAGGTGATATCAATTTTCCTAAAATTCCAGAGAATTTCAATTTGGTTTTTGAGCAGTTTTTTTTGTCTAATATAAATTACACATATCAGATTTGGAAAAAAGGCTAA |
| qacE | unknown[qacE_1_X68232] | Benzylkonium Chloride, Ethidium Bromide, Chlorhexidine, Cetylpyridinium Chloride | 99.65 | 1438 | 1154 | X68232 | ATGAAAGGCTGGCTTTTTCTTGTTATCGCAATAGTTGGCGAAGTAATCGCAACATCCGCATTAAAATCTAGCGAGGGCTTTACTAAGCTTGCCCCTTCCGCCGTTGTCATAATCGGTTATGGCATCGCATTTTATTTTCTTTCTCTGGTTCTGAAATCCATCCCTGTCGGTGTTGCTTATGCAGTCTGGTCGGGACTCGGCGTCGTCATAATTACAGCCATTGCCTGGTTGCTTCATGGGCAAAAGCTTGATGCGTGGGGCTTTGTAGGTATGGGGCTCATAATT |
| qnrS1 | ciprofloxacin I/R | Ciprofloxacin | 100 | 9633 | 8977 | AB187515 | ATGGAAACCTACAATCATACATATCGGCACCACAACTTTTCACATAAAGACTTAAGTGATCTCACCTTCACCGCTTGCACATTCATTCGCAGCGACTTTCGACGTGCTAACTTGCGTGATACGACATTCGTCAACTGCAAGTTCATTGAACAGGGTGATATCGAAGGCTGCCACTTTGATGTCGCAGATCTTCGTGATGCAAGTTTCCAACAATGCCAACTTGCGATGGCAAACTTCAGTAATGCCAATTGCTACGGTATAGAGTTCCGTGCGTGTGATTTAAAAGGTGCCAACTTTTCCCGAACAAACTTTGCCCATCAAGTGAGTAATCGTATGTACTTTTGCTCAGCATTTATTTCTGGATGTAATCTTTCCTATGCCAATATGGAGAGGGTTTGTTTAGAAAAATGTGAGTTGTTTGAAAATCGCTGGATAGGAACGAACCTAGCGGGTGCATCACTGAAAGAGTCAGACTTAAGTCGAGGTGTTTTTTCCGAAGATGTCTGGGGGCAATTTAGCCTACAGGGTGCCAATTTATGCCACGCCGAACTCGACGGTTTAGATCCCCGCAAAGTCGATACATCAGGTATCAAAATTGCAGCCTGGCAGCAAGAACTGATTCTCGAAGCACTGGGTATTGTTGTTTATCCTGACTAA |
| sul1 | sulfisoxazole | Sulfamethoxazole | 100 | 1097 | 258 | U12338 | ATGGTGACGGTGTTCGGCATTCTGAATCTCACCGAGGACTCCTTCTTCGATGAGAGCCGGCGGCTAGACCCCGCCGGCGCTGTCACCGCGGCGATCGAAATGCTGCGAGTCGGATCAGACGTCGTGGATGTCGGACCGGCCGCCAGCCATCCGGACGCGAGGCCTGTATCGCCGGCCGATGAGATCAGACGTATTGCGCCGCTCTTAGACGCCCTGTCCGATCAGATGCACCGTGTTTCAATCGACAGCTTCCAACCGGAAACCCAGCGCTATGCGCTCAAGCGCGGCGTGGGCTACCTGAACGATATCCAAGGATTTCCTGACCCTGCGCTCTATCCCGATATTGCTGAGGCGGACTGCAGGCTGGTGGTTATGCACTCAGCGCAGCGGGATGGCATCGCCACCCGCACCGGTCACCTTCGACCCGAAGACGCGCTCGACGAGATTGTGCGGTTCTTCGAGGCGCGGGTTTCCGCCTTGCGACGGAGCGGGGTCGCTGCCGACCGGCTCATCCTCGATCCGGGGATGGGATTTTTCTTGAGCCCCGCACCGGAAACATCGCTGCACGTGCTGTCGAACCTTCAAAAGCTGAAGTCGGCGTTGGGGCTTCCGCTATTGGTCTCGGTGTCGCGGAAATCCTTCTTGGGCGCCACCGTTGGCCTTCCTGTAAAGGATCTGGGTCCAGCGAGCCTTGCGGCGGAACTTCACGCGATCGGCAATGGCGCTGACTACGTCCGCACCCACGCGCCTGGAGATCTGCGAAGCGCAATCACCTTCTCGGAAACCCTCGCGAAATTTCGCAGTCGCGACGCCAGAGACCGAGGGTTAGATCATGCCTAG |
| sul2 | sulfisoxazole | Sulfamethoxazole | 100 | 2601 | 1786 | HQ840942 | ATGAATAAATCGCTCATCATTTTCGGCATCGTCAACATAACCTCGGACAGTTTCTCCGATGGAGGCCGGTATCTGGCGCCAGACGCAGCCATTGCGCAGGCGCGTAAGCTGATGGCCGAGGGGGCAGATGTGATCGACCTCGGTCCGGCATCCAGCAACCCCGACGCCGCGCCTGTTTCGTCCGACACAGAAATCGAGCGTATCGCGCCGGTGCTGGACGCGCTCAAGGCAGATGGCATTCCCGTCTCGCTCGACAGTTATCAACCCGCGACGCAAGCCTATGCCTTGTCGCGTGGTGTGGCCTATCTCAATGATATTCGCGGTTTTCCAGACGCTGCGTTCTATCCGCAATTGGCGAAATCATCTGCCAAACTCGTCGTTATGCATTCGGTGCAAGACGGGCAGGCAGATCGGCGCGAGGCACCCGCTGGCGACATCATGGATCACATTGCGGCGTTCTTTGACGCGCGCATCGCGGCGCTGACGGGTGCCGGTATCAAACGCAACCGCCTTGTCCTTGATCCCGGCATGGGGTTTTTTCTGGGGGCTGCTCCCGAAACCTCGCTCTCGGTGCTGGCGCGGTTCGATGAATTGCGGCTGCGCTTCGATTTGCCGGTGCTTCTGTCTGTTTCGCGCAAATCCTTTCTGCGCGCGCTCACAGGCCGTGGTCCGGGGGATGTCGGGGCCGCGACACTCGCTGCAGAGCTTGCCGCCGCCGCAGGTGGAGCTGACTTCATCCGCACACACGAGCCGCGCCCCTTGCGCGACGGGCTGGCGGTATTGGCGGCGCTAAAAGAAACCGCAAGAATTCGTTAA |

**Table 30:** Antibiotic resistance genes in 54E

| **Gene** | **Predicted Phenotype** | **CGE Predicted Phenotype** | **%Identity** | **Start** | **End** | **Accession** | **Sequence** |
| --- | --- | --- | --- | --- | --- | --- | --- |
| aph(3'')-Ib | streptomycin | Streptomycin | 99.88 | 1648 | 845 | AF321551 | TTGAATCGAACTAATATTTTTTTGGGTGAATCGCATTCTGACTGGTTGCCTGTCAGAGGCGGAGAATCTGGTGATTTTGTTTTTCGACGTGGTGACGGGCATGCCTTCGCGAAAATCGCACCTGCTTCCCGCCGCGGTGAGCTCGCTGGAGAGCGTGACCGCCTCATTTGGCTCAAAGGTCGAGGTGTGGCTTGCCCCGAGGTCATCAACTGGCAGGAGGAACAGGAGGGTGCATGCTTGGTGATAACGGCAATTCCGGGAGTACCGGCGGCTGATCTGTCTGGAGCGGATTTGCTCAAAGCGTGGCCGTCAATGGGGCAGCAACTTGGCGCTGTTCACAGCCTATCGGTTGATCAATGTCCGTTTGAGCGCAGGCTGTCGCGAATGTTCGGACGCGCCGTTGATGTGGTGTCCCGCAATGCCGTCAATCCCGACTTCTTACCGGACGAGGACAAGAGTACGCCGCAGCTCGATCTTTTGGCTCGTGTCGAACGAGAGCTACCGGTGCGGCTCGACCAAGAGCGCACCGATATGGTTGTTTGCCATGGTGATCCCTGCATGCCGAACTTCATGGTGGACCCTAAAACTCTTCAATGCACGGGTCTGATCGACCTTGGGCGGCTCGGAACAGCAGATCGCTATGCCGATTTGGCACTCATGATTGCTAACGCCGAAGAGAACTGGGCAGCGCCAGATGAAGCAGAGCGCGCCTTCGCTGTCCTATTCAATGTATTGGGGATCGAAGCCCCCGACCGCGAACGCCTTGCCTTCTATCTGCGATTGGACCCTCTGACTTGGGGTTGA |
| aph(6)-Id | kanamycin | Streptomycin | 100 | 845 | 9 | M28829 | ATGTTCATGCCGCCTGTTTTTCCTGCTCATTGGCACGTTTCGCAACCTGTTCTCATTGCGGACACCTTTTCCAGCCTCGTTTGGAAAGTTTCATTGCCAGACGGGACTCCTGCAATCGTCAAGGGATTGAAACCTATAGAAGACATTGCTGATGAACTGCGCGGGGCCGACTATCTGGTATGGCGCAATGGGAGGGGAGCAGTCCGGTTGCTCGGTCGTGAGAACAATCTGATGTTGCTCGAATATGCCGGGGAGCGAATGCTCTCTCACATCGTTGCCGAGCACGGCGACTACCAGGCGACCGAAATTGCAGCGGAACTAATGGCGAAGCTGTATGCCGCATCTGAGGAACCCCTGCCTTCTGCCCTTCTCCCGATCCGGGATCGCTTTGCAGCTTTGTTTCAGCGGGCGCGCGATGATCAAAACGCAGGTTGTCAAACTGACTACGTCCACGCGGCGATTATAGCCGATCAAATGATGAGCAATGCCTCGGAACTGCGTGGGCTACATGGCGATCTGCATCATGAAAACATCATGTTCTCCAGTCGCGGCTGGCTGGTGATAGATCCCGTCGGTCTGGTCGGTGAAGTGGGCTTTGGCGCCGCCAATATGTTCTACGATCCGGCTGACAGAGACGACCTTTGTCTCGATCCTAGACGCATTGCACAGATGGCGGACGCATTCTCTCGTGCGCTGGACGTCGATCCGCGTCGCCTGCTCGACCAGGCGTACGCTTATGGGTGCCTTTCCGCAGCTTGGAACGCGGATGGAGAAGAGGAGCAACGCGATCTAGCTATCGCGGCCGCGATCAAGCAGGTGCGACAGACGTCATACTAG |
| blaCTX-M-15 | ampicillin, ceftriaxone | Amoxicillin, Ampicillin, Aztreonam, Cefepime, Cefotaxime, Ceftazidime, Ceftriaxone, Piperacillin, Ticarcillin | 100 | 3831 | 4706 | AY044436 | ATGGTTAAAAAATCACTGCGCCAGTTCACGCTGATGGCGACGGCAACCGTCACGCTGTTGTTAGGAAGTGTGCCGCTGTATGCGCAAACGGCGGACGTACAGCAAAAACTTGCCGAATTAGAGCGGCAGTCGGGAGGCAGACTGGGTGTGGCATTGATTAACACAGCAGATAATTCGCAAATACTTTATCGTGCTGATGAGCGCTTTGCGATGTGCAGCACCAGTAAAGTGATGGCCGCGGCCGCGGTGCTGAAGAAAAGTGAAAGCGAACCGAATCTGTTAAATCAGCGAGTTGAGATCAAAAAATCTGACCTTGTTAACTATAATCCGATTGCGGAAAAGCACGTCAATGGGACGATGTCACTGGCTGAGCTTAGCGCGGCCGCGCTACAGTACAGCGATAACGTGGCGATGAATAAGCTGATTGCTCACGTTGGCGGCCCGGCTAGCGTCACCGCGTTCGCCCGACAGCTGGGAGACGAAACGTTCCGTCTCGACCGTACCGAGCCGACGTTAAACACCGCCATTCCGGGCGATCCGCGTGATACCACTTCACCTCGGGCAATGGCGCAAACTCTGCGGAATCTGACGCTGGGTAAAGCATTGGGCGACAGCCAACGGGCGCAGCTGGTGACATGGATGAAAGGCAATACCACCGGTGCAGCGAGCATTCAGGCTGGACTGCCTGCTTCCTGGGTTGTGGGGGATAAAACCGGCAGCGGTGGCTATGGCACCACCAACGATATCGCGGTGATCTGGCCAAAAGATCGTGCGCCGCTGATTCTGGTCACTTACTTCACCCAGCCTCAACCTAAGGCAGAAAGCCGTCGCGATGTATTAGCGTCGGCGGCTAAAATCGTCACCGACGGTTTGTAA |
| blaTEM-1B | ampicillin | Amoxicillin, Ampicillin, Cephalothin, Piperacillin, Ticarcillin | 100 | 1009 | 149 | AY458016 | ATGAGTATTCAACATTTTCGTGTCGCCCTTATTCCCTTTTTTGCGGCATTTTGCCTTCCTGTTTTTGCTCACCCAGAAACGCTGGTGAAAGTAAAAGATGCTGAAGATCAGTTGGGTGCACGAGTGGGTTACATCGAACTGGATCTCAACAGCGGTAAGATCCTTGAGAGTTTTCGCCCCGAAGAACGTTTTCCAATGATGAGCACTTTTAAAGTTCTGCTATGTGGTGCGGTATTATCCCGTGTTGACGCCGGGCAAGAGCAACTCGGTCGCCGCATACACTATTCTCAGAATGACTTGGTTGAGTACTCACCAGTCACAGAAAAGCATCTTACGGATGGCATGACAGTAAGAGAATTATGCAGTGCTGCCATAACCATGAGTGATAACACTGCTGCCAACTTACTTCTGACAACGATCGGAGGACCGAAGGAGCTAACCGCTTTTTTGCACAACATGGGGGATCATGTAACTCGCCTTGATCGTTGGGAACCGGAGCTGAATGAAGCCATACCAAACGACGAGCGTGACACCACGATGCCTGCAGCAATGGCAACAACGTTGCGCAAACTATTAACTGGCGAACTACTTACTCTAGCTTCCCGGCAACAATTAATAGACTGGATGGAGGCGGATAAAGTTGCAGGACCACTTCTGCGCTCGGCCCTTCCGGCTGGCTGGTTTATTGCTGATAAATCTGGAGCCGGTGAGCGTGGGTCTCGCGGTATCATTGCAGCACTGGGGCCAGATGGTAAGCCCTCCCGTATCGTAGTTATCTACACGACGGGGAGTCAGGCAACTATGGATGAACGAAATAGACAGATCGCTGAGATAGGTGCCTCACTGATTAAGCATTGGTAA |
| catA1 | chloramphenicol | Chloramphenicol | 99.85 | 299 | 958 | V00622 | ATGGAGAAAAAAATCACTGGATATACCACCGTTGATATATCCCAATGGCATCGTAAAGAACATTTTGAGGCATTTCAGTCAGTTGCTCAATGTACCTATAACCAGACCGTTCAGCTGGATATTACGGCCTTTTTAAAGACCGTAAAGAAAAATAAGCACAAGTTTTATCCGGCCTTTATTCACATTCTTGCCCGCCTGATGAATGCTCATCCGGAATTCCGTATGGCAATGAAAGACGGTGAGCTGGTGATATGGGATAGTGTTCACCCTTGTTACACCGTTTTCCATGAGCAAACTGAAACGTTTTCATCGCTCTGGAGTGAATACCACGACGATTTCCGGCAGTTTCTACACATATATTCGCAAGATGTGGCGTGTTACGGTGAAAACCTGGCCTATTTCCCTAAAGGGTTTATTGAGAATATGTTTTTCGTCTCAGCCAATCCCTGGGTGAGTTTCACCAGTTTTGATTTAAACGTGGCCAATATGGACAACTTCTTCGCCCCCGTTTTCACCATGGGCAAATATTATACGCAAGGCGACAAGGTGCTGATGCCGCTGGCGATTCAGGTTCATCATGCCGTTTGTGATGGCTTCCATGTCGGCAGAATGCTTAATGAATTACAACAGTACTGCGATGAGTGGCAGGGCGGGGCGTAA |
| dfrA7 | trimethoprim | Trimethoprim | 100 | 6982 | 7455 | AB161450 | TTGAAAATTTCATTGATTTCTGCAACGTCAGAAAATGGCGTAATCGGTAATGGCCCTGATATCCCATGGTCAGCAAAAGGTGAGCAGTTACTCTTTAAAGCGCTCACATATAATCAGTGGCTCCTTGTTGGAAGGAAAACATTTGACTCTATGGGTGTTCTTCCAAATCGAAAATATGCAGTAGTGTCGAGGAAAGGAATTTCAAGCTCAAATGAAAATGTATTAGTCTTTCCTTCAATAGAAATCGCTTTGCAAGAACTATCGAAAATTACAGATCATTTATATGTCTCTGGTGGCGGTCAAATCTACAATAGTCTTATTGAAAAAGCAGATATAATTCATTTGTCTACTGTTCACGTTGAGGTTGAAGGTGATATCAATTTTCCTAAAATTCCAGAGAATTTCAATTTGGTTTTTGAGCAGTTTTTTTTGTCTAATATAAATTACACATATCAGATTTGGAAAAAAGGCTAA |
| qacE | unknown[qacE_1_X68232] | Benzylkonium Chloride, Ethidium Bromide, Chlorhexidine, Cetylpyridinium Chloride | 100 | 7685 | 7966 | X68232 | ATGAAAGGCTGGCTTTTTCTTGTTATCGCAATAGTTGGCGAAGTAATCGCAACATCCGCATTAAAATCTAGCGAGGGCTTTACTAAGCTTGCCCCTTCCGCCGTTGTCATAATCGGTTATGGCATCGCATTTTATTTTCTTTCTCTGGTTCTGAAATCCATCCCTGTCGGTGTTGCTTATGCAGTCTGGTCGGGACTCGGCGTCGTCATAATTACAGCCATTGCCTGGTTGCTTCATGGGCAAAAGCTTGATGCGTGGGGCTTTGTAGGTATGGGGCTCATA |
| qnrS1 | ciprofloxacin I/R | Ciprofloxacin | 100 | 9347 | 10003 | AB187515 | ATGGAAACCTACAATCATACATATCGGCACCACAACTTTTCACATAAAGACTTAAGTGATCTCACCTTCACCGCTTGCACATTCATTCGCAGCGACTTTCGACGTGCTAACTTGCGTGATACGACATTCGTCAACTGCAAGTTCATTGAACAGGGTGATATCGAAGGCTGCCACTTTGATGTCGCAGATCTTCGTGATGCAAGTTTCCAACAATGCCAACTTGCGATGGCAAACTTCAGTAATGCCAATTGCTACGGTATAGAGTTCCGTGCGTGTGATTTAAAAGGTGCCAACTTTTCCCGAACAAACTTTGCCCATCAAGTGAGTAATCGTATGTACTTTTGCTCAGCATTTATTTCTGGATGTAATCTTTCCTATGCCAATATGGAGAGGGTTTGTTTAGAAAAATGTGAGTTGTTTGAAAATCGCTGGATAGGAACGAACCTAGCGGGTGCATCACTGAAAGAGTCAGACTTAAGTCGAGGTGTTTTTTCCGAAGATGTCTGGGGGCAATTTAGCCTACAGGGTGCCAATTTATGCCACGCCGAACTCGACGGTTTAGATCCCCGCAAAGTCGATACATCAGGTATCAAAATTGCAGCCTGGCAGCAAGAACTGATTCTCGAAGCACTGGGTATTGTTGTTTATCCTGACTAA |
| sul1 | sulfisoxazole | Sulfamethoxazole | 100 | 8026 | 8865 | U12338 | ATGGTGACGGTGTTCGGCATTCTGAATCTCACCGAGGACTCCTTCTTCGATGAGAGCCGGCGGCTAGACCCCGCCGGCGCTGTCACCGCGGCGATCGAAATGCTGCGAGTCGGATCAGACGTCGTGGATGTCGGACCGGCCGCCAGCCATCCGGACGCGAGGCCTGTATCGCCGGCCGATGAGATCAGACGTATTGCGCCGCTCTTAGACGCCCTGTCCGATCAGATGCACCGTGTTTCAATCGACAGCTTCCAACCGGAAACCCAGCGCTATGCGCTCAAGCGCGGCGTGGGCTACCTGAACGATATCCAAGGATTTCCTGACCCTGCGCTCTATCCCGATATTGCTGAGGCGGACTGCAGGCTGGTGGTTATGCACTCAGCGCAGCGGGATGGCATCGCCACCCGCACCGGTCACCTTCGACCCGAAGACGCGCTCGACGAGATTGTGCGGTTCTTCGAGGCGCGGGTTTCCGCCTTGCGACGGAGCGGGGTCGCTGCCGACCGGCTCATCCTCGATCCGGGGATGGGATTTTTCTTGAGCCCCGCACCGGAAACATCGCTGCACGTGCTGTCGAACCTTCAAAAGCTGAAGTCGGCGTTGGGGCTTCCGCTATTGGTCTCGGTGTCGCGGAAATCCTTCTTGGGCGCCACCGTTGGCCTTCCTGTAAAGGATCTGGGTCCAGCGAGCCTTGCGGCGGAACTTCACGCGATCGGCAATGGCGCTGACTACGTCCGCACCCACGCGCCTGGAGATCTGCGAAGCGCAATCACCTTCTCGGAAACCCTCGCGAAATTTCGCAGTCGCGACGCCAGAGACCGAGGGTTAGATCATGCCTAG |
| sul2 | sulfisoxazole | Sulfamethoxazole | 100 | 2524 | 1709 | HQ840942 | ATGAATAAATCGCTCATCATTTTCGGCATCGTCAACATAACCTCGGACAGTTTCTCCGATGGAGGCCGGTATCTGGCGCCAGACGCAGCCATTGCGCAGGCGCGTAAGCTGATGGCCGAGGGGGCAGATGTGATCGACCTCGGTCCGGCATCCAGCAACCCCGACGCCGCGCCTGTTTCGTCCGACACAGAAATCGAGCGTATCGCGCCGGTGCTGGACGCGCTCAAGGCAGATGGCATTCCCGTCTCGCTCGACAGTTATCAACCCGCGACGCAAGCCTATGCCTTGTCGCGTGGTGTGGCCTATCTCAATGATATTCGCGGTTTTCCAGACGCTGCGTTCTATCCGCAATTGGCGAAATCATCTGCCAAACTCGTCGTTATGCATTCGGTGCAAGACGGGCAGGCAGATCGGCGCGAGGCACCCGCTGGCGACATCATGGATCACATTGCGGCGTTCTTTGACGCGCGCATCGCGGCGCTGACGGGTGCCGGTATCAAACGCAACCGCCTTGTCCTTGATCCCGGCATGGGGTTTTTTCTGGGGGCTGCTCCCGAAACCTCGCTCTCGGTGCTGGCGCGGTTCGATGAATTGCGGCTGCGCTTCGATTTGCCGGTGCTTCTGTCTGTTTCGCGCAAATCCTTTCTGCGCGCGCTCACAGGCCGTGGTCCGGGGGATGTCGGGGCCGCGACACTCGCTGCAGAGCTTGCCGCCGCCGCAGGTGGAGCTGACTTCATCCGCACACACGAGCCGCGCCCCTTGCGCGACGGGCTGGCGGTATTGGCGGCGCTAAAAGAAACCGCAAGAATTCGTTAA |

**Table 31:** Antibiotic resistance genes in 55E genome

| **Gene** | **Predicted Phenotype** | **CGE Predicted Phenotype** | **%Identity** | **Start** | **End** | **Accession** | **Sequence** |
| --- | --- | --- | --- | --- | --- | --- | --- |
| aac(2')-Ia | Gentamicin | Gentamicin, Tobramycin, Dibekacin, Netilmicin | 99.81 | 24654 | 25190 | L06156 | ATGGGCATAGAATACCGCAGTCTGCATACCAGCCAATTGACACTGAGTGAAAAAGAAGCGCTTTACGATTTATTAATTGAAGGTTTTGAAGGCGATTTTTCGCATGACGATTTCGCGCACACTTTAGGTGGAATGCACGTCATGGCTTTTGATCAACAAAAATTGGTTGGTCATGTTGCAATTATTCAACGCCATATGGCCCTAGATAATACGCCTATCTCTGTAGGGTATGTTGAAGCGATGGTAGTTGAACAAAGTTATCGTCGCCAAGGTATTGGGCGGCAATTGATGCTGCAAACCAATAAAATTATAGCTTCGTGTTATCAATTAGGGCTGCTGTCGGCTTCAGATGATGGACAAAAATTGTATCATTCGGTCGGATGGCAAATCTGGAAAGGTAAGTTGTTTGAATTGAAACAAGGGAGCTATATCCGTTCTATTGAAGAAGAAGGCGGAGTCATGGGCTGGAAAGCGGATGGTGAGGTTGATTTTACCGCTTCGCTTTACTGTGATTTTCGTGGCGGTGATCAGTGGTAA |
| aac(6')-Ib-cr | unknown[aac(6')-Ib-cr_1_DQ303918] | Tobramycin, Dibekacin, Amikacin, Sisomicin, Netilmicin, Fluoroquinolone, Ciprofloxacin | 99.64 | 162 | 724 | DQ303918 | ACAGCATCGTGACCAACAGCAACGATTCCGTCACACTGCGCCTCATGACTGAGCATGACCTTGCGATGCTCTATGAGTGGCTAAATCGATCTCATATCGTCGAGTGGTGGGGCGGAGAAGAAGCACGCCCGACACTTGCTGACGTACAGGAACAGTACTTGCCAAGCGTTTTAGCGCAAGAGTCCGTCACTCCATACATTGCAATGCTGAATGGAGAGCCGATTGGGTATGCCCAGTCGTACGTTGCTCTTGGAAGCGGGGACGGATGGTGGGAAGAAGAAACCGATCCAGGAGTACGCGGAATAGACCAGTTACTGGCGAATGCATCACAACTGGGCAAAGGCTTGGGAACCAAGCTGGTTCGAGCTCTGGTTGAGTTGCTGTTCAATGATCCCGAGGTCACCAAGATCCAAACGGACCCGTCGCCGAGCAACTTGCGAGCGATCCGATGCTACGAGAAAGCGGGGTTTGAGAGGCAAGGTACCGTAACCACCCCAGATGGTCCAGCCGTGTACATGGTTCAAACACGCCAGGCATTCGAGCGAACACGCAGTGATGCCTAA |
| aac(6')-Ib3 | amikacin, tobramycin | Amikacin, Tobramycin | 100 | 170 | 724 | X60321 | GTGACCAACAGCAACGATTCCGTCACACTGCGCCTCATGACTGAGCATGACCTTGCGATGCTCTATGAGTGGCTAAATCGATCTCATATCGTCGAGTGGTGGGGCGGAGAAGAAGCACGCCCGACACTTGCTGACGTACAGGAACAGTACTTGCCAAGCGTTTTAGCGCAAGAGTCCGTCACTCCATACATTGCAATGCTGAATGGAGAGCCGATTGGGTATGCCCAGTCGTACGTTGCTCTTGGAAGCGGGGACGGATGGTGGGAAGAAGAAACCGATCCAGGAGTACGCGGAATAGACCAGTTACTGGCGAATGCATCACAACTGGGCAAAGGCTTGGGAACCAAGCTGGTTCGAGCTCTGGTTGAGTTGCTGTTCAATGATCCCGAGGTCACCAAGATCCAAACGGACCCGTCGCCGAGCAACTTGCGAGCGATCCGATGCTACGAGAAAGCGGGGTTTGAGAGGCAAGGTACCGTAACCACCCCAGATGGTCCAGCCGTGTACATGGTTCAAACACGCCAGGCATTCGAGCGAACACGCAGTGATGCCTAA |
| aadA2 | streptomycin | Spectinomycin, Streptomycin | 100 | 922 | 131 | JQ364967 | ATGAGGGAAGCGGTGACCATCGAAATTTCGAACCAACTATCAGAGGTGCTAAGCGTCATTGAGCGCCATCTGGAATCAACGTTGCTGGCCGTGCATTTGTACGGCTCCGCAGTGGATGGCGGCCTGAAGCCATACAGCGATATTGATTTGTTGGTTACTGTGGCCGTAAAGCTTGATGAAACGACGCGGCGAGCATTGCTCAATGATCTTATGGAGGCTTCGGCTTTCCCTGGCGAGAGCGAGACGCTCCGCGCTATAGAAGTCACCCTTGTCGTGCATGACGACATCATCCCGTGGCGTTATCCGGCTAAGCGCGAGCTGCAATTTGGAGAATGGCAGCGCAATGACATTCTTGCGGGTATCTTCGAGCCAGCCATGATCGACATTGATCTAGCTATCCTGCTTACAAAAGCAAGAGAACATAGCGTTGCCTTGGTAGGTCCGGCAGCGGAGGAATTCTTTGACCCGGTTCCTGAACAGGATCTATTCGAGGCGCTGAGGGAAACCTTGAAGCTATGGAACTCGCAGCCCGACTGGGCCGGCGATGAGCGAAATGTAGTGCTTACGTTGTCCCGCATTTGGTACAGCGCAATAACCGGCAAAATCGCGCCGAAGGATGTCGCTGCCGACTGGGCAATAAAACGCCTACCTGCCCAGTATCAGCCCGTCTTACTTGAAGCTAAGCAAGCTTATCTGGGACAAAAAGAAGATCACTTGGCCTCACGCGCAGATCACTTGGAAGAATTTATTCGCTTTGTGAAAGGCGAGATCATCAAGTCAGTTGGTAAATGA |
| aph(3'')-Ib | streptomycin | Streptomycin | 100 | 4320 | 3517 | AF321551 | TTGAATCGAACTAATATTTTTTTTGGTGAATCGCATTCTGACTGGTTGCCTGTCAGAGGCGGAGAATCTGGTGATTTTGTTTTTCGACGTGGTGACGGGCATGCCTTCGCGAAAATCGCACCTGCTTCCCGCCGCGGTGAGCTCGCTGGAGAGCGTGACCGCCTCATTTGGCTCAAAGGTCGAGGTGTGGCTTGCCCCGAGGTCATCAACTGGCAGGAGGAACAGGAGGGTGCATGCTTGGTGATAACGGCAATTCCGGGAGTACCGGCGGCTGATCTGTCTGGAGCGGATTTGCTCAAAGCGTGGCCGTCAATGGGGCAGCAACTTGGCGCTGTTCACAGCCTATCGGTTGATCAATGTCCGTTTGAGCGCAGGCTGTCGCGAATGTTCGGACGCGCCGTTGATGTGGTGTCCCGCAATGCCGTCAATCCCGACTTCTTACCGGACGAGGACAAGAGTACGCCGCAGCTCGATCTTTTGGCTCGTGTCGAACGAGAGCTACCGGTGCGGCTCGACCAAGAGCGCACCGATATGGTTGTTTGCCATGGTGATCCCTGCATGCCGAACTTCATGGTGGACCCTAAAACTCTTCAATGCACGGGTCTGATCGACCTTGGGCGGCTCGGAACAGCAGATCGCTATGCCGATTTGGCACTCATGATTGCTAACGCCGAAGAGAACTGGGCAGCGCCAGATGAAGCAGAGCGCGCCTTCGCTGTCCTATTCAATGTATTGGGGATCGAAGCCCCCGACCGCGAACGCCTTGCCTTCTATCTGCGATTGGACCCTCTGACTTGGGGTTGA |
| aph(3')-Ia | Kanamycin | Neomycin, Kanamycin, Lividomycin, Paromomycin, Ribostamycin | 100 | 1029 | 214 | V00359 | ATGAGCCATATTCAACGGGAAACGTCTTGCTCGAGGCCGCGATTAAATTCCAACATGGATGCTGATTTATATGGGTATAAATGGGCTCGCGATAATGTCGGGCAATCAGGTGCGACAATCTATCGATTGTATGGGAAGCCCGATGCGCCAGAGTTGTTTCTGAAACATGGCAAAGGTAGCGTTGCCAATGATGTTACAGATGAGATGGTCAGACTAAACTGGCTGACGGAATTTATGCCTCTTCCGACCATCAAGCATTTTATCCGTACTCCTGATGATGCATGGTTACTCACCACTGCGATCCCCGGGAAAACAGCATTCCAGGTATTAGAAGAATATCCTGATTCAGGTGAAAATATTGTTGATGCGCTGGCAGTGTTCCTGCGCCGGTTGCATTCGATTCCTGTTTGTAATTGTCCTTTTAACAGCGATCGCGTATTTCGTCTCGCTCAGGCGCAATCACGAATGAATAACGGTTTGGTTGATGCGAGTGATTTTGATGACGAGCGTAATGGCTGGCCTGTTGAACAAGTCTGGAAAGAAATGCATAAGCTTTTGCCATTCTCACCGGATTCAGTCGTCACTCATGGTGATTTCTCACTTGATAACCTTATTTTTGACGAGGGGAAATTAATAGGTTGTATTGATGTTGGACGAGTCGGAATCGCAGACCGATACCAGGATCTTGCCATCCTATGGAACTGCCTCGGTGAGTTTTCTCCTTCATTACAGAAACGGCTTTTTCAAAAATATGGTATTGATAATCCTGATATGAATAAATTGCAGTTTCATTTGATGCTCGATGAGTTTTTCTAA |
| aph(3')-VIa | Kanamycin | Kanamycin, Neomycin, Paromomycin, Ribostamycin, Butirosin, Gentamicin, Amikacin | 99.87 | 58 | 837 | X07753 | ATGGAATTGCCCAATATTATTCAACAATTTATCGGAAACAGCGTTTTAGAGCCAAATAAAATTGGTCAGTCGCCATCGGATGTTTATTCTTTTAATCGAAATAATGAAACTTTTTTTCTTAAGCGATCTAGCACTTTATATACAGAGACCACATACAGTGTCTCTCGTGAAGCGAAAATGTTGAGTTGGCTCTCTGAGAAATTAAAGGTGCCTGAACTCATCATGACTTTTCAGGATGAGCAGTTTGAATTCATGATCACTAAAGCGATCAATGCAAAACCAATTTCAGCGCTTTTTTTAACAGACCAAGAATTGCTTGCTATCTATAAGGAGGCACTCAATCTGTTAAATTCAATTGCTATTATTGATTGTCCATTTATTTCAAACATTGATCATCGGTTAAAAGAGTCAAAATTTTTTATTGATAACCAACTCCTTGACGATATAGATCAAGATGATTTGGACACTGAATTATGGGGAGACCATAAAACTTACCTAAGTCTATGGAATGAGTTAACCGAGACTCGTGTTGAAGAAAGATTGGTTTTTTCTCATGGCGATATCACGGATAGTAATATTTTTATAGATAAATTCAATGAAATTTATTTTTTAGATCTTGGTCGTGCTGGGTTAGCAGATGAATTTGTAGATATATCCTTTGTTGAACGTTGCCTAAGAGAGGATGCATCGGAGGAAACTGCGAAAATATTTTTAAAGCATTTAAAAAATGATAGACCTGACAAAAGGAATTATTTTTTAAAACTTGATGAATTGAATTGA |
| aph(6)-Id | Kanamycin | Streptomycin | 100 | 3517 | 2681 | M28829 | ATGTTCATGCCGCCTGTTTTTCCTGCTCATTGGCACGTTTCGCAACCTGTTCTCATTGCGGACACCTTTTCCAGCCTCGTTTGGAAAGTTTCATTGCCAGACGGGACTCCTGCAATCGTCAAGGGATTGAAACCTATAGAAGACATTGCTGATGAACTGCGCGGGGCCGACTATCTGGTATGGCGCAATGGGAGGGGAGCAGTCCGGTTGCTCGGTCGTGAGAACAATCTGATGTTGCTCGAATATGCCGGGGAGCGAATGCTCTCTCACATCGTTGCCGAGCACGGCGACTACCAGGCGACCGAAATTGCAGCGGAACTAATGGCGAAGCTGTATGCCGCATCTGAGGAACCCCTGCCTTCTGCCCTTCTCCCGATCCGGGATCGCTTTGCAGCTTTGTTTCAGCGGGCGCGCGATGATCAAAACGCAGGTTGTCAAACTGACTACGTCCACGCGGCGATTATAGCCGATCAAATGATGAGCAATGCCTCGGAACTGCGTGGGCTACATGGCGATCTGCATCATGAAAACATCATGTTCTCCAGTCGCGGCTGGCTGGTGATAGATCCCGTCGGTCTGGTCGGTGAAGTGGGCTTTGGCGCCGCCAATATGTTCTACGATCCGGCTGACAGAGACGACCTTTGTCTCGATCCTAGACGCATTGCACAGATGGCGGACGCATTCTCTCGTGCGCTGGACGTCGATCCGCGTCGCCTGCTCGACCAGGCGTACGCTTATGGGTGCCTTTCCGCAGCTTGGAACGCGGATGGAGAAGAGGAGCAACGCGATCTAGCTATCGCGGCCGCGATCAAGCAGGTGCGACAGACGTCATACTAG |
| blaCMY-16 | ampicillin, amoxicillin/clavulanic acid, cefoxitin, ceftriaxone | Amoxicillin, Amoxicillin+Clavulanic acid, Ampicillin, Ampicillin+Clavulanic acid, Cefotaxime, Cefoxitin, Ceftazidime, Piperacillin, Piperacillin+Tazobactam, Ticarcillin, Ticarcillin+Clavulanic acid | 99.39 | 2328 | 1177 | AJ781421 | ATGATGAAAAAATCGTTATGCTGCGCTCTGCTGCTGACAGCCTCTTTCTCCACATTTGCTGCCGCAAAAACAGAACAACAGATTGCCGATATCGTTAATCGCACCATCACCCCGTTGATGCAGGAGCAGGCTATTCCGGGTATGGCCGTTGCCGTTATCTACCAGGGAAAACCCTATTATTTCACCTGGGGTAAAGCCGATATCGCCAATAACCACCCAGTCACGCAGCAAACGCTGTTTGAGCTAGGATCGGTTAGTAAGACGTTTAACGGCGTGTTGGGCGGCGATGCTATCGCCCGCGGCGAAATTAAGCTCAGCGATCCGGTCACGAAATACTGGCCAGAACTGACAGGCAAACAGTGGCAGGGTATCCGCCTGCTGCACTTAGCCACCTATACGGCAGGCGGCCTACCGCTGCAGATCCCCGATGACGTTAGGGATAAAGCCGCATTACTGCATTTTTATCAAAACTGGCAGCCGCAATGGACTCCGGGCGCTAAGCTACTTTACTCTAACTCCAGCATTGGTCTGTTTGGCGCGCTGGCGGTGAAACCCTCAGGAATGAGTTACGAAGAGGCAATGACCAGACGCGTCCTGCAACCATTAAAACTGGCGCATACCTGGATTACGGTTCCGCAGAACGAACAAAAAGATTATGCCCGGGGCTATCGCGAAGGGAAGCCCGTGCCCGTACACGTTTCTCCGGGACAACTTGACGCCGAAGCCTATGGCGTGAAATCCAGCGTTATTGATATGGCCCGCTGGGTTCAGGCCAACATGGATGCCAGCCACGTTCAGGAGAAAACGCTCCAGCAGGGCATTGCGCTTGCGCAGTCTCGCTACTGGCGTATTGGCGATATGTACCAGGGATTAGGCTGGGAGATGCTGAACTGGCCGCTGAAAGCTGATTCGATCATCAACGGCAGCGACAGCAAAGTGGCATTGGCAGCGCTTCCCGCCGTTGAGGTAAACCCGCCCGCCCCCGCAGTGAAAGCCTCATGGGTGCATAAAACGGGCTCCACTGGTGGATTTGGCAGCTACGTAGCCTTCGTTCCAGAAAAAAACCTTGGCATCGTGATGCTGGCAAACAAAAGCTATCCTAACCCTGTCCGTGTCGAGGCGGCCTGGCGCATTCTTGAAAAGCTGCAATAA |
| blaCTX-M-15 | ampicillin, ceftriaxone | Amoxicillin, Ampicillin, Aztreonam, Cefepime, Cefotaxime, Ceftazidime, Ceftriaxone, Piperacillin, Ticarcillin | 100 | 14039 | 13164 | AY044436 | ATGGTTAAAAAATCACTGCGCCAGTTCACGCTGATGGCGACGGCAACCGTCACGCTGTTGTTAGGAAGTGTGCCGCTGTATGCGCAAACGGCGGACGTACAGCAAAAACTTGCCGAATTAGAGCGGCAGTCGGGAGGCAGACTGGGTGTGGCATTGATTAACACAGCAGATAATTCGCAAATACTTTATCGTGCTGATGAGCGCTTTGCGATGTGCAGCACCAGTAAAGTGATGGCCGCGGCCGCGGTGCTGAAGAAAAGTGAAAGCGAACCGAATCTGTTAAATCAGCGAGTTGAGATCAAAAAATCTGACCTTGTTAACTATAATCCGATTGCGGAAAAGCACGTCAATGGGACGATGTCACTGGCTGAGCTTAGCGCGGCCGCGCTACAGTACAGCGATAACGTGGCGATGAATAAGCTGATTGCTCACGTTGGCGGCCCGGCTAGCGTCACCGCGTTCGCCCGACAGCTGGGAGACGAAACGTTCCGTCTCGACCGTACCGAGCCGACGTTAAACACCGCCATTCCGGGCGATCCGCGTGATACCACTTCACCTCGGGCAATGGCGCAAACTCTGCGGAATCTGACGCTGGGTAAAGCATTGGGCGACAGCCAACGGGCGCAGCTGGTGACATGGATGAAAGGCAATACCACCGGTGCAGCGAGCATTCAGGCTGGACTGCCTGCTTCCTGGGTTGTGGGGGATAAAACCGGCAGCGGTGGCTATGGCACCACCAACGATATCGCGGTGATCTGGCCAAAAGATCGTGCGCCGCTGATTCTGGTCACTTACTTCACCCAGCCTCAACCTAAGGCAGAAAGCCGTCGCGATGTATTAGCGTCGGCGGCTAAAATCGTCACCGACGGTTTGTAA |
| blaNDM-1 | ampicillin, amoxicillin/clavulanic acid, cefoxitin, ceftriaxone, meropenem | Amoxicillin, Amoxicillin+Clavulanic acid, Ampicillin, Ampicillin+Clavulanic acid, Cefepime, Cefixime, Cefotaxime, Cefoxitin, Ceftazidime, Ertapenem, Imipenem, Meropenem, Piperacillin, Piperacillin+Tazobactam, Temocillin | 100 | 17258 | 16446 | FN396876 | ATGGAATTGCCCAATATTATGCACCCGGTCGCGAAGCTGAGCACCGCATTAGCCGCTGCATTGATGCTGAGCGGGTGCATGCCCGGTGAAATCCGCCCGACGATTGGCCAGCAAATGGAAACTGGCGACCAACGGTTTGGCGATCTGGTTTTCCGCCAGCTCGCACCGAATGTCTGGCAGCACACTTCCTATCTCGACATGCCGGGTTTCGGGGCAGTCGCTTCCAACGGTTTGATCGTCAGGGATGGCGGCCGCGTGCTGGTGGTCGATACCGCCTGGACCGATGACCAGACCGCCCAGATCCTCAACTGGATCAAGCAGGAGATCAACCTGCCGGTCGCGCTGGCGGTGGTGACTCACGCGCATCAGGACAAGATGGGCGGTATGGACGCGCTGCATGCGGCGGGGATTGCGACTTATGCCAATGCGTTGTCGAACCAGCTTGCCCCGCAAGAGGGGATGGTTGCGGCGCAACACAGCCTGACTTTCGCCGCCAATGGCTGGGTCGAACCAGCAACCGCGCCCAACTTTGGCCCGCTCAAGGTATTTTACCCCGGCCCCGGCCACACCAGTGACAATATCACCGTTGGGATCGACGGCACCGACATCGCTTTTGGTGGCTGCCTGATCAAGGACAGCAAGGCCAAGTCGCTCGGCAATCTCGGTGATGCCGACACTGAGCACTACGCCGCGTCAGCGCGCGCGTTTGGTGCGGCGTTCCCCAAGGCCAGCATGATCGTGATGAGCCATTCCGCCCCCGATAGCCGCGCCGCAATCACTCATACGGCCCGCATGGCCGACAAGCTGCGCTGA |
| blaOXA-23 | ampicillin, meropenem | Imipenem, Meropenem | 100 | 1964 | 1143 | AY795964 | ATGAATAAATATTTTACTTGCTATGTGGTTGCTTCTCTTTTTCTTTCTGGTTGTACGGTTCAGCATAATTTAATAAATGAAACCCCGAGTCAGATTGTTCAAGGACATAATCAGGTGATTCATCAATACTTTGATGAAAAAAACACCTCAGGTGTGCTGGTTATTCAAACAGATAAAAAAATTAATCTATATGGTAATGCTCTAAGCCGCGCAAATACAGAATATGTGCCAGCCTCTACATTTAAAATGTTGAATGCCCTGATCGGATTGGAGAACCAGAAAACGGATATTAATGAAATATTTAAATGGAAGGGCGAGAAAAGGTCATTTACCGCTTGGGAAAAAGACATGACACTAGGAGAAGCCATGAAGCTTTCTGCAGTCCCAGTCTATCAGGAACTTGCGCGACGTATCGGTCTTGATCTCATGCAAAAAGAAGTAAAACGTATTGGTTTCGGTAATGCTGAAATTGGACAGCAGGTTGATAATTTCTGGTTGGTAGGACCATTAAAGGTTACGCCTATTCAAGAGGTAGAGTTTGTTTCCCAATTAGCACATACACAGCTTCCATTTAGTGAAAAAGTGCAGGCTAATGTAAAAAATATGCTTCTTTTAGAAGAGAGTAATGGCTACAAAATTTTTGGAAAGACTGGTTGGGCAATGGATATAAAACCACAAGTGGGCTGGTTGACCGGCTGGGTTGAGCAGCCAGATGGAAAAATTGTCGCTTTTGCATTAAATATGGAAATGCGGTCAGAAATGCCGGCATCTATACGTAATGAATTATTGATGAAATCATTAAAACAGCTGAATATTATTTAA |
| blaTEM-1B | Ampicillin | Amoxicillin, Ampicillin, Cephalothin, Piperacillin, Ticarcillin | 100 | 437 | 1297 | AY458016 | ATGAGTATTCAACATTTTCGTGTCGCCCTTATTCCCTTTTTTGCGGCATTTTGCCTTCCTGTTTTTGCTCACCCAGAAACGCTGGTGAAAGTAAAAGATGCTGAAGATCAGTTGGGTGCACGAGTGGGTTACATCGAACTGGATCTCAACAGCGGTAAGATCCTTGAGAGTTTTCGCCCCGAAGAACGTTTTCCAATGATGAGCACTTTTAAAGTTCTGCTATGTGGTGCGGTATTATCCCGTGTTGACGCCGGGCAAGAGCAACTCGGTCGCCGCATACACTATTCTCAGAATGACTTGGTTGAGTACTCACCAGTCACAGAAAAGCATCTTACGGATGGCATGACAGTAAGAGAATTATGCAGTGCTGCCATAACCATGAGTGATAACACTGCTGCCAACTTACTTCTGACAACGATCGGAGGACCGAAGGAGCTAACCGCTTTTTTGCACAACATGGGGGATCATGTAACTCGCCTTGATCGTTGGGAACCGGAGCTGAATGAAGCCATACCAAACGACGAGCGTGACACCACGATGCCTGCAGCAATGGCAACAACGTTGCGCAAACTATTAACTGGCGAACTACTTACTCTAGCTTCCCGGCAACAATTAATAGACTGGATGGAGGCGGATAAAGTTGCAGGACCACTTCTGCGCTCGGCCCTTCCGGCTGGCTGGTTTATTGCTGATAAATCTGGAGCCGGTGAGCGTGGGTCTCGCGGTATCATTGCAGCACTGGGGCCAGATGGTAAGCCCTCCCGTATCGTAGTTATCTACACGACGGGGAGTCAGGCAACTATGGATGAACGAAATAGACAGATCGCTGAGATAGGTGCCTCACTGATTAAGCATTGGTAA |
| catA1 | chloramphenicol | Chloramphenicol | 99.85 | 299 | 958 | V00622 | ATGGAGAAAAAAATCACTGGATATACCACCGTTGATATATCCCAATGGCATCGTAAAGAACATTTTGAGGCATTTCAGTCAGTTGCTCAATGTACCTATAACCAGACCGTTCAGCTGGATATTACGGCCTTTTTAAAGACCGTAAAGAAAAATAAGCACAAGTTTTATCCGGCCTTTATTCACATTCTTGCCCGCCTGATGAATGCTCATCCGGAATTCCGTATGGCAATGAAAGACGGTGAGCTGGTGATATGGGATAGTGTTCACCCTTGTTACACCGTTTTCCATGAGCAAACTGAAACGTTTTCATCGCTCTGGAGTGAATACCACGACGATTTCCGGCAGTTTCTACACATATATTCGCAAGATGTGGCGTGTTACGGTGAAAACCTGGCCTATTTCCCTAAAGGGTTTATTGAGAATATGTTTTTCGTCTCAGCCAATCCCTGGGTGAGTTTCACCAGTTTTGATTTAAACGTGGCCAATATGGACAACTTCTTCGCCCCCGTTTTCACCATGGGCAAATATTATACGCAAGGCGACAAGGTGCTGATGCCGCTGGCGATTCAGGTTCATCATGCCGTTTGTGATGGCTTCCATGTCGGCAGAATGCTTAATGAATTACAACAGTACTGCGATGAGTGGCAGGGCGGGGCGTAA |
| catA3 | chloramphenicol | Chloramphenicol | 100 | 2486 | 3127 | X07848 | ATGAACTATACAAAATTTGATGTAAAAAATTGGGTTCGCCGTGAGCATTTTGAGTTTTATCGGCATCGTTTACCATGTGGTTTTAGCTTAACAAGCAAAATTGATATCACGACGTTAAAAAAGTCATTGGATGATTCAGCGTATAAGTTTTATCCGGTAATGATCTATCTGATTGCTCAGGCCGTGAATCAATTTGATGAGTTGAGAATGGCGATAAAAGATGATGAATTGATCGTATGGGATTCAGTCGACCCACAATTCACCGTATTCCATCAAGAAACAGAGACATTTTCAGCACTGAGTTGCCCATACTCATCCGATATTGATCAATTTATGGTGAATTATTTATCGGTAATGGAACGTTATAAAAGTGATACCAAGTTATTTCCTCAAGGGGTAACACCAGAAAATCATTTAAATATTTCAGCATTACCTTGGGTTAATTTTGATAGCTTTAATTTAAATGTTGCTAATTTTACCGATTATTTTGCACCCATTATAACAATGGCAAAATATCAGCAAGAAGGGGATAGACTGTTATTGCCGCTCTCAGTACAGGTTCATCATGCAGTTTGTGATGGCTTCCATGTTGCACGCTTTATTAATCGGCTACAAGAGTTGTGTAACAGTAAATTAAAATAA |
| dfrA12 | trimethoprim | Trimethoprim | 100 | 1827 | 1330 | AM040708 | ATGAACTCGGAATCAGTACGCATTTATCTCGTTGCTGCGATGGGAGCCAATCGGGTTATTGGCAATGGTCCTAATATCCCCTGGAAAATTCCGGGTGAGCAGAAGATTTTTCGCAGACTCACTGAGGGAAAAGTCGTTGTCATGGGGCGAAAGACCTTTGAGTCTATCGGCAAGCCTCTACCGAACCGTCACACATTGGTAATCTCACGCCAAGCTAACTACCGCGCCACTGGCTGCGTAGTTGTTTCAACGCTGTCGCACGCTATCGCTTTGGCATCCGAACTCGGCAATGAACTCTACGTCGCGGGCGGAGCTGAGATATACACTCTGGCACTACCTCACGCCCACGGCGTGTTTCTATCTGAGGTACATCAAACCTTCGAGGGTGACGCCTTCTTCCCAATGCTCAACGAAACAGAATTCGAGCTTGTCTCAACCGAAACCATTCAAGCTGTAATTCCGTACACCCACTCCGTTTATGCGCGTCGAAACGGCTAA |
| dfrA7 | trimethoprim | Trimethoprim | 100 | 91 | 564 | AB161450 | TTGAAAATTTCATTGATTTCTGCAACGTCAGAAAATGGCGTAATCGGTAATGGCCCTGATATCCCATGGTCAGCAAAAGGTGAGCAGTTACTCTTTAAAGCGCTCACATATAATCAGTGGCTCCTTGTTGGAAGGAAAACATTTGACTCTATGGGTGTTCTTCCAAATCGAAAATATGCAGTAGTGTCGAGGAAAGGAATTTCAAGCTCAAATGAAAATGTATTAGTCTTTCCTTCAATAGAAATCGCTTTGCAAGAACTATCGAAAATTACAGATCATTTATATGTCTCTGGTGGCGGTCAAATCTACAATAGTCTTATTGAAAAAGCAGATATAATTCATTTGTCTACTGTTCACGTTGAGGTTGAAGGTGATATCAATTTTCCTAAAATTCCAGAGAATTTCAATTTGGTTTTTGAGCAGTTTTTTTTGTCTAATATAAATTACACATATCAGATTTGGAAAAAAGGCTAA |
| lnu(E) | Lincomycin | Lincomycin | 99.37 | 318 | 1 | KF287643 | TTGGGAAAAAATAATGTCACAGAAAAACATCTATTTTATATTTTAGATTTACTTAAAGACCTCCAAATAACTTATTGGTTAGACGGTGGATGGGGAGTAGATGTACTCACTGGAAAGCAACAGAGAGATCACAGAGACATAGATATCGATTTTGTTTCACAACATACAGACAAATTAGTTAAAAAATTAAAAGAGATTGGATACATCACAGTTGTAGATTGGATGCCTTCCAGAATGGAATTAAAACACGAAGAATACGGATATTTAGATATACATCCCTTAGATTTAAAAAAAGATGGCACAGCAACTCAAGCCGAT |
| mph(A) | erythromycin, azithromycin | Erythromycin, Azithromycin, Spiramycin, Telithromycin | 99.67 | 147 | 1068 | U36578 | ATGACCGTAGTCACGACCGCCGATACCTCCCAACTGTACGCACTTGCAGCCCGACATGGGCTCAAGCTCCATGGCCCGCTGACTGTCAATGAGCTTGGGCTCGACTATAGGATCGTGATCGCCACCGTCGACGATGGACGTCGGTGGGTGCTGCGCATCCCGCGCCGAGCCGAGGTAAGCGCGAAGGTCGAACCAGAGGCGCGGGTGCTGGCAATGCTCAAGAATCGCCTGCCGTTCGCGGTGCCGGACTGGCGCGTGGCCAACGCCGAGCTCGTTGCCTATCCCATGCTCGAAGACTCGACTGCGATGGTCATCCAGCCTGGTTCGTCCACGCCCGACTGGGTCGTGCCGCAGGACTCGGAGGTCTTCGCGGAGAGCTTCGCGACCGCGCTCGCCGCCCTGCATGCCGTCCCCATTTCCGCCGCCGTGGATGCGGGGATGCTCATCCGTACACCGACGCAGGCCCGTCAGAAGGTGGCCGACGACGTTGACCGCGTCCGACGCGAGTTCGTGGTGAACGACAAGCGCCTCCACCGGTGGCAGCGCTGGCTCGACGACGATTCGTCGTGGCCAGATTTCTCCGTGGTGGTGCATGGCGATCTCTACGTGGGCCATGTGCTCATCGACAACACGGAGCGCGTCAGCGGGATGATCGACTGGAGCGAGGCCCGCGTTGATGACCCTGCCATCGACATGGCCGCGCACCTTATGGTCTTTGGTGAAGAGGGGCTCGCGAAGCTCCTCCTCACGTATGAAGCGGCCGGTGGCCGGGTGTGGCCGCGGCTCGCCCACCACATCGCGGAGCGCCTTGCGTTCGGGGCGGTCACCTACGCACTCTTCGCCCTCGACTCGGGTAACGAAGAGTACCTCGCTGCGGCGAAGGCGCAGCTCGCCGCAGCGGAATGAGCGAACGTCGATATAG |
| mph(E) | erythromycin, azithromycin | Erythromycin | 100 | 5917 | 5033 | DQ839391 | ATGACAATTCAAGATATTCAATCACTTGCTGAAGCACACGGCTTGTTGCTTACGGACAAAATGAATTTCAATGAAATGGGCATTGATTTTAAGGTCGTTTTTGCTCTTGATACAAAGGGGCAACAATGGTTGCTGCGTATTCCTCGTCGTGATGGCATGAGGGAACAAATCAAGAAAGAAAAACGCATTTTAGAATTGGTAAAAAAACATCTTTCTGTAGAGGTTCCTGATTGGAGAATTTCATCTACAGAATTAGTGGCTTATCCCATACTTAAAGATAATCCTGTTTTAAATTTGGATGCTGAAACCTATGAAATAATTTGGAATATGGACAAAGATAGCCCGAAATACATAACATCTTTGGCAAAAACCTTATTTGAAATCCATAGTATTCCTGAAAAAGAAGTTCGGGAAAATGATTTGAAAATTATGAAACCTTCAGATTTAAGACCTGAAATAGCAAACAATTTGCAGTTAGTAAAATCTGAAATTGGTATAAGTGAGCAATTGGAAACCCGCTACAGAAAATGGTTGGATAATGATGTTCTATGGGCAGATTTCACCCAATTTATACATGGCGATTTATATGCTGGGCATGTACTAGCTTCAAAGGATGGAGCTGTTTCAGGCGTTATTGATTGGTCAACAGCCCATATAGATGACCCAGCGATTGATTTTGCTGGGCATGTAACTTTGTTTGGAGAAGAAAGCCTCAAAACTCTAATCATCGAGTATGAAAAACTAGGGGGTAAAGTTTGGAATAAACTATATGAACAGACTTTAGAAAGAGCAGCGGCCTCTCCTTTGATGTATGGTTTATTTGCCTTAGAAACTCAAAATGAAAGCCTTATCGTTGGAGCAAAAGCTCAGTTGGGAGTTATATAA |
| msr(E) | erythromycin, azithromycin | Erythromycin, Azithromycin, Quinupristin, Pristinamycin IA, Virginiamycin S | 100 | 6894 | 5973 | FR751518 | GCGTCAAAAAGAAGAAGAGCGACAACACCAAGCCGTAGAATATGAGCTGATGATGAAGGAACGGGAGCGATTAGAATCTGCTGTGCAAGAAAAACGCCAGCAAGCTAATCGATTAGACAATAAGAAAAAAGGAGAAAAATCCAAAAACTCTACCGAAAGTGCTGGACGACTTGGGCATGCAAAAATGACTGGCACCAAGCAAAGAAAACTGTATCAGGCAGCTAAGAGTATGGAAAAGCGTTTGGCTGCATTAGAAGATATTCAAGCACCAGAGCATTTGCGTTCTATTCGTTTTCGTCAAAGTTCAGCCCTAGAACTGCACAATAAGTTCCCGATTACGGCAGATGGTCTGAGCTTAAAATTTGGTAGCCGTACTATCTTTGATGACGCTAACTTTATAATACCGCTTGGCGCTAAAGTCGCTATAACTGGATCGAATGGAACAGGGAAAACGTCCTTGTTAAAAATGATATCAGAACGTGCTGATGGATTAACCATATCTCCAAAAGCTGAAATTGGCTACTTTACACAAACAGGATATAAATTTAACACGCATAAATCTGTGCTCTCCTTTATGCAGGAAGAGTGCGAGTACACAGTTGCGGAAATTCGTGCAGTATTGGCTTCAATGGGGATCGGAGCGAATGATATTCAAAAAAACTTATCCGACTTATCGGGAGGTGAAATCATCAAACTGCTTTTATCCAAAATGCTTTTAGGAAAATATAATATTTTGCTTATGGATGAACCAGGAAACTATCTTGACCTAAAAAGTATTGCCGCATTAGAAACAATGATGAAGTCCTATGCAGGAACTATTATCTTCGTATCTCATGACAAGCAATTGGTCGATAATATTGCTGACATTATCTACGAGATCAAAGACCACAAAATCATCAAGACTTTTGAGAGAGATTGTTAA |
| qacE | unknown[qacE_1_X68232] | Benzylkonium Chloride, Ethidium Bromide, Chlorhexidine, Cetylpyridinium Chloride | 99.65 | 1363 | 1079 | X68232 | ATGAAAGGCTGGCTTTTTCTTGTTATCGCAATAGTTGGCGAAGTAATCGCAACATCCGCATTAAAATCTAGCGAGGGCTTTACTAAGCTTGCCCCTTCCGCCGTTGTCATAATCGGTTATGGCATCGCATTTTATTTTCTTTCTCTGGTTCTGAAATCCATCCCTGTCGGTGTTGCTTATGCAGTCTGGTCGGGACTCGGCGTCGTCATAATTACAGCCATTGCCTGGTTGCTTCATGGGCAAAAGCTTGATGCGTGGGGCTTTGTAGGTATGGGGCTCATAATT |
| qnrD1 | unknown[qnrD1_1_FJ228229] | Ciprofloxacin | 100 | 732 | 1376 | FJ228229 | ATGGAAAAGCACTTTATCAATGAAAAGTTTTCACGAGATCAATTTACGGGGAATAGAGTTAAAAATATTGCCTTTTCAAATTGTGATTTTTCAGGGGTTGATTTAACTGATACTGAATTTGTTGATTGTAGTTTTTACGACAGGAATAGCTTGGAAGGGTGTGATTTTAATAGAGCCAAACTAAAAAACGCTAGCTTTAAAAGCTGCGATTTATCAATGAGTAATTTTAAAAACATTAGCGCCTTAGGTCTTGAAATTAGTGAGTGTTTAGCTCAAGGAGCTGATTTTCGAGGGGCTAATTTTATGAATATGATAACTACAAGGTCATGGTTTTGTAGTGCTTATATAACCAAGACAAATCTTAGTTACGCTAATTTTTCTAGAGTCATATTAGAAAAGTGCGAACTGTGGGAAAATCGCTGGAATGGCACTGTGATAACTGGCGCCGTGTTTCGTGGCTCCGATCTTTCTTGTGGGGAGTTTTCATCGTTTGATTGGTCTTTGGCTGATTTTACTGGTTGTGATTTAACGGGTGGGGCGCTTGGCGAGCTTGATGCAAGGCGAATTAATTTAGATGGAGTGAAGTTGGATGGAGAGCAGGCGCTTCAGCTTGTTGAGAGTTTAGGTGTTATTGTTCACCGATAA |
| qnrS1 | ciprofloxacin I/R | Ciprofloxacin | 100 | 8523 | 7867 | AB187515 | ATGGAAACCTACAATCATACATATCGGCACCACAACTTTTCACATAAAGACTTAAGTGATCTCACCTTCACCGCTTGCACATTCATTCGCAGCGACTTTCGACGTGCTAACTTGCGTGATACGACATTCGTCAACTGCAAGTTCATTGAACAGGGTGATATCGAAGGCTGCCACTTTGATGTCGCAGATCTTCGTGATGCAAGTTTCCAACAATGCCAACTTGCGATGGCAAACTTCAGTAATGCCAATTGCTACGGTATAGAGTTCCGTGCGTGTGATTTAAAAGGTGCCAACTTTTCCCGAACAAACTTTGCCCATCAAGTGAGTAATCGTATGTACTTTTGCTCAGCATTTATTTCTGGATGTAATCTTTCCTATGCCAATATGGAGAGGGTTTGTTTAGAAAAATGTGAGTTGTTTGAAAATCGCTGGATAGGAACGAACCTAGCGGGTGCATCACTGAAAGAGTCAGACTTAAGTCGAGGTGTTTTTTCCGAAGATGTCTGGGGGCAATTTAGCCTACAGGGTGCCAATTTATGCCACGCCGAACTCGACGGTTTAGATCCCCGCAAAGTCGATACATCAGGTATCAAAATTGCAGCCTGGCAGCAAGAACTGATTCTCGAAGCACTGGGTATTGTTGTTTATCCTGACTAA |
| rmtC | amikacin, gentamicin, kanamycin, streptomycin | Amikacin, Gentamicin, Tobramycin, Kanamycin, Arbekacin, Sisomicin, Isepamicin | 100 | 107 | 952 | AB194779 | ATGAAAACCAACGATAATTATATCGAAGAAGTAACAGCCAAAGTACTCACAAGTGGTAAATACTCCACACTTTATCCACCAACTGTACGACGTGTAACTGAGAGGCTTTTCGATCGATACCCTCCCAAGCAGCTAGAGAAGGAGGTTCGCAAGAAATTGCATCAAGCATATGGTGCTTATATTGGTGGGATCGATGGGAAAAGGTTGGAGAAGAAGATTGAGAAGATAATTCATGAGATACCAAATCCAACTACGGATGAAGCAACTCGTACGGAGTGGGAAAAAGAGATCTGCCTGAAAATATTGAACTTGCACACTTCAACAAATGAGCGAACGGTGGCTTACGATGAGCTTTACCAAAAGATCTTTGAGGTAACAGGGGTTCCAACAAGTATCACCGATGCAGGTTGCGCTTTGAATCCATTTTCTTTTCCATTCTTTACGGAGGCTGGCATGCTTGGGCAATACATAGGTTTCGATCTTGATAAAGGTATGATCGAAGCGATCGAACACTCGTTGAGAACGCTTAACGCCCCAGAGGGTATTGTTGTCAAACAGGGAGATATATTATCCGATCCGTCAGGCGAGAGTGATCTTCTACTTATGTTCAAGCTATATACTCTACTCGATCGGCAGGAAGAGGCCTCTGGTTTGAAAATTCTTCAAGAGTGGAAATACAAAAATGCTGTGATCTCTTTTCCTATTAAAACTATAAGTGGGAGAGATGTTGGGATGGAAGAGAATTACACTGTTAAGTTCGAGAATGATCTTGTTGGGTCAGATCTGAGAATCATGCAAAAATTGAAATTAGGAAACGAGATGTATTTTATCGTATCGAGATTGTAA |
| sul1 | sulfisoxazole | Sulfamethoxazole | 100 | 1022 | 183 | U12338 | ATGGTGACGGTGTTCGGCATTCTGAATCTCACCGAGGACTCCTTCTTCGATGAGAGCCGGCGGCTAGACCCCGCCGGCGCTGTCACCGCGGCGATCGAAATGCTGCGAGTCGGATCAGACGTCGTGGATGTCGGACCGGCCGCCAGCCATCCGGACGCGAGGCCTGTATCGCCGGCCGATGAGATCAGACGTATTGCGCCGCTCTTAGACGCCCTGTCCGATCAGATGCACCGTGTTTCAATCGACAGCTTCCAACCGGAAACCCAGCGCTATGCGCTCAAGCGCGGCGTGGGCTACCTGAACGATATCCAAGGATTTCCTGACCCTGCGCTCTATCCCGATATTGCTGAGGCGGACTGCAGGCTGGTGGTTATGCACTCAGCGCAGCGGGATGGCATCGCCACCCGCACCGGTCACCTTCGACCCGAAGACGCGCTCGACGAGATTGTGCGGTTCTTCGAGGCGCGGGTTTCCGCCTTGCGACGGAGCGGGGTCGCTGCCGACCGGCTCATCCTCGATCCGGGGATGGGATTTTTCTTGAGCCCCGCACCGGAAACATCGCTGCACGTGCTGTCGAACCTTCAAAAGCTGAAGTCGGCGTTGGGGCTTCCGCTATTGGTCTCGGTGTCGCGGAAATCCTTCTTGGGCGCCACCGTTGGCCTTCCTGTAAAGGATCTGGGTCCAGCGAGCCTTGCGGCGGAACTTCACGCGATCGGCAATGGCGCTGACTACGTCCGCACCCACGCGCCTGGAGATCTGCGAAGCGCAATCACCTTCTCGGAAACCCTCGCGAAATTTCGCAGTCGCGACGCCAGAGACCGAGGGTTAGATCATGCCTAG |
| sul2 | sulfisoxazole | Sulfamethoxazole | 100 | 848 | 33 | AY034138 | ATGAATAAATCGCTCATCATTTTCGGCATCGTCAACATAACCTCGGACAGTTTCTCCGATGGAGGCCGGTATCTGGCGCCAGACGCAGCCATTGCGCAGGCGCGTAAGCTGATGGCCGAGGGGGCAGATGTGATCGACCTCGGTCCGGCATCCAGCAATCCCGACGCCGCGCCTGTTTCGTCCGACACAGAAATCGCGCGTATCGCGCCGGTGCTGGACGCGCTCAAGGCAGATGGCATTCCCGTCTCGCTCGACAGTTATCAACCCGCGACGCAAGCCTATGCCTTGTCGCGTGGTGTGGCCTATCTCAATGATATTCGCGGTTTTCCAGACGCTGCGTTCTATCCGCAATTGGCGAAATCATCTGCCAAACTCGTCGTTATGCATTCGGTGCAAGACGGGCAGGCAGATCGGCGCGAGGCACCCGCTGGCGACATCATGGATCACATTGCGGCGTTCTTTGACGCGCGCATCGCGGCGCTGACGGGTGCCGGTATCAAACGCAACCGCCTTGTCCTTGATCCCGGCATGGGGTTTTTTCTGGGGGCTGCTCCCGAAACCTCGCTCTCGGTGCTGGCGCGGTTCGATGAATTGCGGCTGCGCTTCGATTTGCCGGTGCTTCTGTCTGTTTCGCGCAAATCCTTTCTGCGCGCGCTCACAGGCCGTGGTCCGGGGGATGTCGGGGCCGCGACACTCGCTGCAGAGCTTGCCGCCGCCGCAGGTGGAGCTGACTTCATCCGCACACACGAGCCGCGCCCCTTGCGCGACGGGCTGGCGGTATTGGCGGCGCTGAAAGAAACCGCAAGAATTCGTTAA |
| tet(39) | tetracycline | Doxycycline, Tetracycline | 100 | 2885 | 4006 | KT346360 | ATGCCGATCTTGCCTGAATTATTACGGTCATTGGCTGGAGCTGAAGCAGGCGGTGTTCACTATGGTGCTTTATTAGCTGTGTATGCTCTGATGCAGTTCATTTTTGCACCTATCCTTGGAGCGTTGAGTGACCGATTTGGACGTCGACCTGTATTAATTATTTCAATTGCTGGTGCAACGGCTGATTATCTCCTAATGGCTGCTGCTCCTTCTCTATTGTGGCTATATATTGGTCGTATTTTTGCGGGAATTACAGGTGCCAACATGGCTGTTGCAACAGCTTATGTTTCAGATATTACTCCAGCCCATGAGCGTGCAAAAAGGTTTGGTCTCCTTGGAGCTGTCTTTGGTATTGGGTTTATAGCGGGTCCGGTAATAGGTGGAGTTTTGGGTGAATGGAACTTACATGCACCGTTCTTTGCTGCTGCTTTTATGAATGGGATTAATTTAATAATGACAGCAGTCTTATTAAAAGAATCAAAACACAGCAATAAAATGACTGAGAAGGTTCAGGAGCAATCAATATTAAAGAAATTATCCTATTTGATCACTCAACCTAATATGGCTCCATTGCTTGGTATCTTTTTAATTATCACATTGGTTTCACAAGTCCCCGCAACTTTATGGGTTATCTATGGGCAGGATCGTTATGGCTGGAGTATATTTATTGCAGGTGTTTCCCTTGCTAGTTATGGAATATGCCATTCTATTGCACAGGCTTTTGCTATCGCCCCTATGGTAAAGAGGTTTGGAGAGAAAAATACGTTGTTATGTGGAATAGCTTGCGATGCAATTGGTTTACTTCTTTTATCTATTGCTGTTGAAGAATGGGTGCCTTTTGCGTTGTTACCATTGTTTGCCCTTGGTGGAGTAGCCGTTCCTGCTTTGCAAGCAATGATGTCCAGAGGTATTAGTGATGAAAGACAAGGTGAATTACAAGGGCTATTAAGCAGTTTTAATAGTCTGGGGGCTATAATTGGTCCTGTATTAGTTACTAGCCTCTATTTTATGACTCAGGCATCAGCTCCTGGAATGGTATGGGCATTAGCTGCAATACTTTATGTAATCACCCTACCCTTATTGCTTAAGTATCGCCTGAATAAATATTCTGGAGTTCCATAA |
| tet(B) | tetracycline | Doxycycline, Tetracycline, Minocycline | 100 | 168006 | 166801 | AP000342 | ATGAATAGTTCGACAAAGATCGCATTGGTAATTACGTTACTCGATGCCATGGGGATTGGCCTTATCATGCCAGTCTTGCCAACGTTATTACGTGAATTTATTGCTTCGGAAGATATCGCTAACCACTTTGGCGTATTGCTTGCACTTTATGCGTTAATGCAGGTTATCTTTGCTCCTTGGCTTGGAAAAATGTCTGACCGATTTGGTCGGCGCCCAGTGCTGTTGTTGTCATTAATAGGCGCATCGCTGGATTACTTATTGCTGGCTTTTTCAAGTGCGCTTTGGATGCTGTATTTAGGCCGTTTGCTTTCAGGGATCACAGGAGCTACTGGGGCTGTCGCGGCATCGGTCATTGCCGATACCACCTCAGCTTCTCAACGCGTGAAGTGGTTCGGTTGGTTAGGGGCAAGTTTTGGGCTTGGTTTAATAGCGGGGCCTATTATTGGTGGTTTTGCAGGAGAGATTTCACCGCATAGTCCCTTTTTTATCGCTGCGTTGCTAAATATTGTCACTTTCCTTGTGGTTATGTTTTGGTTCCGTGAAACCAAAAATACACGTGATAATACAGATACCGAAGTAGGGGTTGAGACGCAATCGAATTCGGTATACATCACTTTATTTAAAACGATGCCCATTTTGTTGATTATTTATTTTTCAGCGCAATTGATAGGCCAAATTCCCGCAACGGTGTGGGTGCTATTTACCGAAAATCGTTTTGGATGGAATAGCATGATGGTTGGCTTTTCATTAGCGGGTCTTGGTCTTTTACACTCAGTATTCCAAGCCTTTGTGGCAGGAAGAATAGCCACTAAATGGGGCGAAAAAACGGCAGTACTGCTCGGATTTATTGCAGATAGTAGTGCATTTGCCTTTTTAGCGTTTATATCTGAAGGTTGGTTAGTTTTCCCTGTTTTAATTTTATTGGCTGGTGGTGGGATCGCTTTACCTGCATTACAGGGAGTGATGTCTATCCAAACAAAGAGTCATCAGCAAGGTGCTTTACAGGGATTATTGGTGAGCCTTACCAATGCAACCGGTGTTATTGGCCCATTACTGTTTGCTGTTATTTATAATCATTCACTACCAATTTGGGATGGCTGGATTTGGATTATTGGTTTAGCGTTTTACTGTATTATTATCCTGCTATCGATGACCTTCATGTTAACCCCTCAAGCTCAGGGGAGTAAACAGGAGACAAGTGCTTAG |

**Table 32:** Antibiotic resistance genes in 56E genome

| **Gene** | **Predicted Phenotype** | **CGE Predicted Phenotype** | **%Identity** | **Start** | **End** | **Accession** | **Sequence** |
| --- | --- | --- | --- | --- | --- | --- | --- |
| aac(2')-Ia | gentamicin | Gentamicin, Tobramycin, Dibekacin, Netilmicin | 99.81 | 24384 | 24920 | L06156 | ATGGGCATAGAATACCGCAGTCTGCATACCAGCCAATTGACACTGAGTGAAAAAGAAGCGCTTTACGATTTATTAATTGAAGGTTTTGAAGGCGATTTTTCGCATGACGATTTCGCGCACACTTTAGGTGGAATGCACGTCATGGCTTTTGATCAACAAAAATTGGTTGGTCATGTTGCAATTATTCAACGCCATATGGCCCTAGATAATACGCCTATCTCTGTAGGGTATGTTGAAGCGATGGTAGTTGAACAAAGTTATCGTCGCCAAGGTATTGGGCGGCAATTGATGCTGCAAACCAATAAAATTATAGCTTCGTGTTATCAATTAGGGCTGCTGTCGGCTTCAGATGATGGACAAAAATTGTATCATTCGGTCGGATGGCAAATCTGGAAAGGTAAGTTGTTTGAATTGAAACAAGGGAGCTATATCCGTTCTATTGAAGAAGAAGGCGGAGTCATGGGCTGGAAAGCGGATGGTGAGGTTGATTTTACCGCTTCGCTTTACTGTGATTTTCGTGGCGGTGATCAGTGGTAA |
| aac(6')-Ib-cr | unknown[aac(6')-Ib-cr_1_DQ303918] | Tobramycin, Dibekacin, Amikacin, Sisomicin, Netilmicin, Fluoroquinolone, Ciprofloxacin | 99.64 | 748 | 186 | DQ303918 | ACAGCATCGTGACCAACAGCAACGATTCCGTCACACTGCGCCTCATGACTGAGCATGACCTTGCGATGCTCTATGAGTGGCTAAATCGATCTCATATCGTCGAGTGGTGGGGCGGAGAAGAAGCACGCCCGACACTTGCTGACGTACAGGAACAGTACTTGCCAAGCGTTTTAGCGCAAGAGTCCGTCACTCCATACATTGCAATGCTGAATGGAGAGCCGATTGGGTATGCCCAGTCGTACGTTGCTCTTGGAAGCGGGGACGGATGGTGGGAAGAAGAAACCGATCCAGGAGTACGCGGAATAGACCAGTTACTGGCGAATGCATCACAACTGGGCAAAGGCTTGGGAACCAAGCTGGTTCGAGCTCTGGTTGAGTTGCTGTTCAATGATCCCGAGGTCACCAAGATCCAAACGGACCCGTCGCCGAGCAACTTGCGAGCGATCCGATGCTACGAGAAAGCGGGGTTTGAGAGGCAAGGTACCGTAACCACCCCAGATGGTCCAGCCGTGTACATGGTTCAAACACGCCAGGCATTCGAGCGAACACGCAGTGATGCCTAA |
| aac(6')-Ib3 | amikacin, tobramycin | Amikacin, Tobramycin | 100 | 740 | 186 | X60321 | GTGACCAACAGCAACGATTCCGTCACACTGCGCCTCATGACTGAGCATGACCTTGCGATGCTCTATGAGTGGCTAAATCGATCTCATATCGTCGAGTGGTGGGGCGGAGAAGAAGCACGCCCGACACTTGCTGACGTACAGGAACAGTACTTGCCAAGCGTTTTAGCGCAAGAGTCCGTCACTCCATACATTGCAATGCTGAATGGAGAGCCGATTGGGTATGCCCAGTCGTACGTTGCTCTTGGAAGCGGGGACGGATGGTGGGAAGAAGAAACCGATCCAGGAGTACGCGGAATAGACCAGTTACTGGCGAATGCATCACAACTGGGCAAAGGCTTGGGAACCAAGCTGGTTCGAGCTCTGGTTGAGTTGCTGTTCAATGATCCCGAGGTCACCAAGATCCAAACGGACCCGTCGCCGAGCAACTTGCGAGCGATCCGATGCTACGAGAAAGCGGGGTTTGAGAGGCAAGGTACCGTAACCACCCCAGATGGTCCAGCCGTGTACATGGTTCAAACACGCCAGGCATTCGAGCGAACACGCAGTGATGCCTAA |
| aadA2 | streptomycin | Spectinomycin, Streptomycin | 100 | 972 | 181 | JQ364967 | ATGAGGGAAGCGGTGACCATCGAAATTTCGAACCAACTATCAGAGGTGCTAAGCGTCATTGAGCGCCATCTGGAATCAACGTTGCTGGCCGTGCATTTGTACGGCTCCGCAGTGGATGGCGGCCTGAAGCCATACAGCGATATTGATTTGTTGGTTACTGTGGCCGTAAAGCTTGATGAAACGACGCGGCGAGCATTGCTCAATGATCTTATGGAGGCTTCGGCTTTCCCTGGCGAGAGCGAGACGCTCCGCGCTATAGAAGTCACCCTTGTCGTGCATGACGACATCATCCCGTGGCGTTATCCGGCTAAGCGCGAGCTGCAATTTGGAGAATGGCAGCGCAATGACATTCTTGCGGGTATCTTCGAGCCAGCCATGATCGACATTGATCTAGCTATCCTGCTTACAAAAGCAAGAGAACATAGCGTTGCCTTGGTAGGTCCGGCAGCGGAGGAATTCTTTGACCCGGTTCCTGAACAGGATCTATTCGAGGCGCTGAGGGAAACCTTGAAGCTATGGAACTCGCAGCCCGACTGGGCCGGCGATGAGCGAAATGTAGTGCTTACGTTGTCCCGCATTTGGTACAGCGCAATAACCGGCAAAATCGCGCCGAAGGATGTCGCTGCCGACTGGGCAATAAAACGCCTACCTGCCCAGTATCAGCCCGTCTTACTTGAAGCTAAGCAAGCTTATCTGGGACAAAAAGAAGATCACTTGGCCTCACGCGCAGATCACTTGGAAGAATTTATTCGCTTTGTGAAAGGCGAGATCATCAAGTCAGTTGGTAAATGA |
| aph(3')-Ia | kanamycin | Neomycin, Kanamycin, Lividomycin, Paromomycin, Ribostamycin | 100 | 6782 | 5967 | V00359 | ATGAGCCATATTCAACGGGAAACGTCTTGCTCGAGGCCGCGATTAAATTCCAACATGGATGCTGATTTATATGGGTATAAATGGGCTCGCGATAATGTCGGGCAATCAGGTGCGACAATCTATCGATTGTATGGGAAGCCCGATGCGCCAGAGTTGTTTCTGAAACATGGCAAAGGTAGCGTTGCCAATGATGTTACAGATGAGATGGTCAGACTAAACTGGCTGACGGAATTTATGCCTCTTCCGACCATCAAGCATTTTATCCGTACTCCTGATGATGCATGGTTACTCACCACTGCGATCCCCGGGAAAACAGCATTCCAGGTATTAGAAGAATATCCTGATTCAGGTGAAAATATTGTTGATGCGCTGGCAGTGTTCCTGCGCCGGTTGCATTCGATTCCTGTTTGTAATTGTCCTTTTAACAGCGATCGCGTATTTCGTCTCGCTCAGGCGCAATCACGAATGAATAACGGTTTGGTTGATGCGAGTGATTTTGATGACGAGCGTAATGGCTGGCCTGTTGAACAAGTCTGGAAAGAAATGCATAAGCTTTTGCCATTCTCACCGGATTCAGTCGTCACTCATGGTGATTTCTCACTTGATAACCTTATTTTTGACGAGGGGAAATTAATAGGTTGTATTGATGTTGGACGAGTCGGAATCGCAGACCGATACCAGGATCTTGCCATCCTATGGAACTGCCTCGGTGAGTTTTCTCCTTCATTACAGAAACGGCTTTTTCAAAAATATGGTATTGATAATCCTGATATGAATAAATTGCAGTTTCATTTGATGCTCGATGAGTTTTTCTAA |
| blaCMY-6 | ampicillin, amoxicillin/clavulanic acid, cefoxitin, ceftriaxone | Amoxicillin, Amoxicillin+Clavulanic acid, Ampicillin, Ampicillin+Clavulanic acid, Cefotaxime, Cefoxitin, Ceftazidime, Piperacillin, Piperacillin+Tazobactam, Ticarcillin, Ticarcillin+Clavulanic acid | 100 | 36791 | 35646 | AJ011293 | ATGATGAAAAAATCGTTATGCTGCGCTCTGCTGCTGACAGCCTCTTTCTCCACATTTGCTGCCGCAAAAACAGAACAACAGATTGCCGATATCGTTAATCGCACCATCACCCCGTTGATGCAGGAGCAGGCTATTCCGGGTATGGCCGTTGCCGTTATCTACCAGGGAAAACCCTATTATTTCACCTGGGGTAAAGCCGATATCGCCAATAACCACCCAGTCACGCAGCAAACGCTGTTTGAGCTAGGATCGGTTAGTAAGACGTTTAACGGCGTGTTGGGCGGCGATGCTATCGCCCGCGGCGAAATTAAGCTCAGCGATCCGGTCACGAAATACTGGCCAGAACTGACAGGCAAACAGTGGCAGGGTATCCGCCTGCTGCACTTAGCCACCTATACGGCAGGCGGCCTACCGCTGCAGATCCCCGATGACGTTAGGGATAAAGCCGCATTACTGCATTTTTATCAAAACTGGCAGCCGCAATGGACTCCGGGCGCTAAGCGACTTTACGCTAACTCCAGCATTGGTCTGTTTGGCGCGCTGGCGGTGAAACCCTCAGGAATGAGTTACGAAGAGGCAATGACCAGACGCGTCCTGCAACCATTAAAACTGGCGCATACCTGGATTACGGTTCCGCAGAACGAACAAAAAGATTATGCCTTGGGCTATCGCGAAGGGAAGCCCGTACACGTTTCTCCGGGACAACTTGACGCCGAAGCCTATGGCGTGAAATCCAGCGTTATTGATATGGCCCGCTGGGTTCAGGCCAACATGGATGCCAGCCACGTTCAGGAGAAAACGCTCCAGCAGGGCATTGCGCTTGCGCAGTCTCGCTACTGGCGTATTGGCGATATGTACCAGGGATTAGGCTGGGAGATGCTGAACTGGCCGCTGAAAGCTGATTCGATCATCAACGGCAGCGACAGCAAAGTGGCATTGGCAGCGCTTCCCGCCGTTGAGGTAAACCCGCCCGCCCCCGCAGTGAAAGCCTCATGGGTGCATAAAACGGGCTCCACTGGTGGATTTGGCAGCTACGTAGCCTTCGTTCCAGAAAAAAACCTTGGCATCGTGATGCTGGCAAACAAAAGCTATCCTAACCCTGTCCGTGTCGAGGCGGCCTGGCGCATTCTTGAAAAGCTGCAATAA |
| blaNDM-1 | ampicillin, amoxicillin/clavulanic acid, cefoxitin, ceftriaxone, meropenem | Amoxicillin, Amoxicillin+Clavulanic acid, Ampicillin, Ampicillin+Clavulanic acid, Cefepime, Cefixime, Cefotaxime, Cefoxitin, Ceftazidime, Ertapenem, Imipenem, Meropenem, Piperacillin, Piperacillin+Tazobactam, Temocillin | 100 | 17457 | 16645 | FN396876 | ATGGAATTGCCCAATATTATGCACCCGGTCGCGAAGCTGAGCACCGCATTAGCCGCTGCATTGATGCTGAGCGGGTGCATGCCCGGTGAAATCCGCCCGACGATTGGCCAGCAAATGGAAACTGGCGACCAACGGTTTGGCGATCTGGTTTTCCGCCAGCTCGCACCGAATGTCTGGCAGCACACTTCCTATCTCGACATGCCGGGTTTCGGGGCAGTCGCTTCCAACGGTTTGATCGTCAGGGATGGCGGCCGCGTGCTGGTGGTCGATACCGCCTGGACCGATGACCAGACCGCCCAGATCCTCAACTGGATCAAGCAGGAGATCAACCTGCCGGTCGCGCTGGCGGTGGTGACTCACGCGCATCAGGACAAGATGGGCGGTATGGACGCGCTGCATGCGGCGGGGATTGCGACTTATGCCAATGCGTTGTCGAACCAGCTTGCCCCGCAAGAGGGGATGGTTGCGGCGCAACACAGCCTGACTTTCGCCGCCAATGGCTGGGTCGAACCAGCAACCGCGCCCAACTTTGGCCCGCTCAAGGTATTTTACCCCGGCCCCGGCCACACCAGTGACAATATCACCGTTGGGATCGACGGCACCGACATCGCTTTTGGTGGCTGCCTGATCAAGGACAGCAAGGCCAAGTCGCTCGGCAATCTCGGTGATGCCGACACTGAGCACTACGCCGCGTCAGCGCGCGCGTTTGGTGCGGCGTTCCCCAAGGCCAGCATGATCGTGATGAGCCATTCCGCCCCCGATAGCCGCGCCGCAATCACTCATACGGCCCGCATGGCCGACAAGCTGCGCTGA |
| catA3 | chloramphenicol | Chloramphenicol | 100 | 2536 | 3177 | X07848 | ATGAACTATACAAAATTTGATGTAAAAAATTGGGTTCGCCGTGAGCATTTTGAGTTTTATCGGCATCGTTTACCATGTGGTTTTAGCTTAACAAGCAAAATTGATATCACGACGTTAAAAAAGTCATTGGATGATTCAGCGTATAAGTTTTATCCGGTAATGATCTATCTGATTGCTCAGGCCGTGAATCAATTTGATGAGTTGAGAATGGCGATAAAAGATGATGAATTGATCGTATGGGATTCAGTCGACCCACAATTCACCGTATTCCATCAAGAAACAGAGACATTTTCAGCACTGAGTTGCCCATACTCATCCGATATTGATCAATTTATGGTGAATTATTTATCGGTAATGGAACGTTATAAAAGTGATACCAAGTTATTTCCTCAAGGGGTAACACCAGAAAATCATTTAAATATTTCAGCATTACCTTGGGTTAATTTTGATAGCTTTAATTTAAATGTTGCTAATTTTACCGATTATTTTGCACCCATTATAACAATGGCAAAATATCAGCAAGAAGGGGATAGACTGTTATTGCCGCTCTCAGTACAGGTTCATCATGCAGTTTGTGATGGCTTCCATGTTGCACGCTTTATTAATCGGCTACAAGAGTTGTGTAACAGTAAATTAAAATAA |
| dfrA12 | trimethoprim | Trimethoprim | 100 | 1877 | 1380 | AM040708 | ATGAACTCGGAATCAGTACGCATTTATCTCGTTGCTGCGATGGGAGCCAATCGGGTTATTGGCAATGGTCCTAATATCCCCTGGAAAATTCCGGGTGAGCAGAAGATTTTTCGCAGACTCACTGAGGGAAAAGTCGTTGTCATGGGGCGAAAGACCTTTGAGTCTATCGGCAAGCCTCTACCGAACCGTCACACATTGGTAATCTCACGCCAAGCTAACTACCGCGCCACTGGCTGCGTAGTTGTTTCAACGCTGTCGCACGCTATCGCTTTGGCATCCGAACTCGGCAATGAACTCTACGTCGCGGGCGGAGCTGAGATATACACTCTGGCACTACCTCACGCCCACGGCGTGTTTCTATCTGAGGTACATCAAACCTTCGAGGGTGACGCCTTCTTCCCAATGCTCAACGAAACAGAATTCGAGCTTGTCTCAACCGAAACCATTCAAGCTGTAATTCCGTACACCCACTCCGTTTATGCGCGTCGAAACGGCTAA |
| dfrA7 | trimethoprim | Trimethoprim | 100 | 717 | 244 | AB161450 | TTGAAAATTTCATTGATTTCTGCAACGTCAGAAAATGGCGTAATCGGTAATGGCCCTGATATCCCATGGTCAGCAAAAGGTGAGCAGTTACTCTTTAAAGCGCTCACATATAATCAGTGGCTCCTTGTTGGAAGGAAAACATTTGACTCTATGGGTGTTCTTCCAAATCGAAAATATGCAGTAGTGTCGAGGAAAGGAATTTCAAGCTCAAATGAAAATGTATTAGTCTTTCCTTCAATAGAAATCGCTTTGCAAGAACTATCGAAAATTACAGATCATTTATATGTCTCTGGTGGCGGTCAAATCTACAATAGTCTTATTGAAAAAGCAGATATAATTCATTTGTCTACTGTTCACGTTGAGGTTGAAGGTGATATCAATTTTCCTAAAATTCCAGAGAATTTCAATTTGGTTTTTGAGCAGTTTTTTTTGTCTAATATAAATTACACATATCAGATTTGGAAAAAAGGCTAA |
| qacE | unknown[qacE_1_X68232] | Benzylkonium Chloride, Ethidium Bromide, Chlorhexidine, Cetylpyridinium Chloride | 99.65 | 1363 | 1079 | X68232 | ATGAAAGGCTGGCTTTTTCTTGTTATCGCAATAGTTGGCGAAGTAATCGCAACATCCGCATTAAAATCTAGCGAGGGCTTTACTAAGCTTGCCCCTTCCGCCGTTGTCATAATCGGTTATGGCATCGCATTTTATTTTCTTTCTCTGGTTCTGAAATCCATCCCTGTCGGTGTTGCTTATGCAGTCTGGTCGGGACTCGGCGTCGTCATAATTACAGCCATTGCCTGGTTGCTTCATGGGCAAAAGCTTGATGCGTGGGGCTTTGTAGGTATGGGGCTCATAATT |
| qnrD1 | unknown[qnrD1_1_FJ228229] | Ciprofloxacin | 100 | 3354 | 2710 | FJ228229 | ATGGAAAAGCACTTTATCAATGAAAAGTTTTCACGAGATCAATTTACGGGGAATAGAGTTAAAAATATTGCCTTTTCAAATTGTGATTTTTCAGGGGTTGATTTAACTGATACTGAATTTGTTGATTGTAGTTTTTACGACAGGAATAGCTTGGAAGGGTGTGATTTTAATAGAGCCAAACTAAAAAACGCTAGCTTTAAAAGCTGCGATTTATCAATGAGTAATTTTAAAAACATTAGCGCCTTAGGTCTTGAAATTAGTGAGTGTTTAGCTCAAGGAGCTGATTTTCGAGGGGCTAATTTTATGAATATGATAACTACAAGGTCATGGTTTTGTAGTGCTTATATAACCAAGACAAATCTTAGTTACGCTAATTTTTCTAGAGTCATATTAGAAAAGTGCGAACTGTGGGAAAATCGCTGGAATGGCACTGTGATAACTGGCGCCGTGTTTCGTGGCTCCGATCTTTCTTGTGGGGAGTTTTCATCGTTTGATTGGTCTTTGGCTGATTTTACTGGTTGTGATTTAACGGGTGGGGCGCTTGGCGAGCTTGATGCAAGGCGAATTAATTTAGATGGAGTGAAGTTGGATGGAGAGCAGGCGCTTCAGCTTGTTGAGAGTTTAGGTGTTATTGTTCACCGATAA |
| sul1 | sulfisoxazole | Sulfamethoxazole | 100 | 1022 | 183 | U12338 | ATGGTGACGGTGTTCGGCATTCTGAATCTCACCGAGGACTCCTTCTTCGATGAGAGCCGGCGGCTAGACCCCGCCGGCGCTGTCACCGCGGCGATCGAAATGCTGCGAGTCGGATCAGACGTCGTGGATGTCGGACCGGCCGCCAGCCATCCGGACGCGAGGCCTGTATCGCCGGCCGATGAGATCAGACGTATTGCGCCGCTCTTAGACGCCCTGTCCGATCAGATGCACCGTGTTTCAATCGACAGCTTCCAACCGGAAACCCAGCGCTATGCGCTCAAGCGCGGCGTGGGCTACCTGAACGATATCCAAGGATTTCCTGACCCTGCGCTCTATCCCGATATTGCTGAGGCGGACTGCAGGCTGGTGGTTATGCACTCAGCGCAGCGGGATGGCATCGCCACCCGCACCGGTCACCTTCGACCCGAAGACGCGCTCGACGAGATTGTGCGGTTCTTCGAGGCGCGGGTTTCCGCCTTGCGACGGAGCGGGGTCGCTGCCGACCGGCTCATCCTCGATCCGGGGATGGGATTTTTCTTGAGCCCCGCACCGGAAACATCGCTGCACGTGCTGTCGAACCTTCAAAAGCTGAAGTCGGCGTTGGGGCTTCCGCTATTGGTCTCGGTGTCGCGGAAATCCTTCTTGGGCGCCACCGTTGGCCTTCCTGTAAAGGATCTGGGTCCAGCGAGCCTTGCGGCGGAACTTCACGCGATCGGCAATGGCGCTGACTACGTCCGCACCCACGCGCCTGGAGATCTGCGAAGCGCAATCACCTTCTCGGAAACCCTCGCGAAATTTCGCAGTCGCGACGCCAGAGACCGAGGGTTAGATCATGCCTAG |
| tet(B) | tetracycline | Doxycycline, Tetracycline, Minocycline | 100 | 16426 | 17631 | AP000342 | ATGAATAGTTCGACAAAGATCGCATTGGTAATTACGTTACTCGATGCCATGGGGATTGGCCTTATCATGCCAGTCTTGCCAACGTTATTACGTGAATTTATTGCTTCGGAAGATATCGCTAACCACTTTGGCGTATTGCTTGCACTTTATGCGTTAATGCAGGTTATCTTTGCTCCTTGGCTTGGAAAAATGTCTGACCGATTTGGTCGGCGCCCAGTGCTGTTGTTGTCATTAATAGGCGCATCGCTGGATTACTTATTGCTGGCTTTTTCAAGTGCGCTTTGGATGCTGTATTTAGGCCGTTTGCTTTCAGGGATCACAGGAGCTACTGGGGCTGTCGCGGCATCGGTCATTGCCGATACCACCTCAGCTTCTCAACGCGTGAAGTGGTTCGGTTGGTTAGGGGCAAGTTTTGGGCTTGGTTTAATAGCGGGGCCTATTATTGGTGGTTTTGCAGGAGAGATTTCACCGCATAGTCCCTTTTTTATCGCTGCGTTGCTAAATATTGTCACTTTCCTTGTGGTTATGTTTTGGTTCCGTGAAACCAAAAATACACGTGATAATACAGATACCGAAGTAGGGGTTGAGACGCAATCGAATTCGGTATACATCACTTTATTTAAAACGATGCCCATTTTGTTGATTATTTATTTTTCAGCGCAATTGATAGGCCAAATTCCCGCAACGGTGTGGGTGCTATTTACCGAAAATCGTTTTGGATGGAATAGCATGATGGTTGGCTTTTCATTAGCGGGTCTTGGTCTTTTACACTCAGTATTCCAAGCCTTTGTGGCAGGAAGAATAGCCACTAAATGGGGCGAAAAAACGGCAGTACTGCTCGGATTTATTGCAGATAGTAGTGCATTTGCCTTTTTAGCGTTTATATCTGAAGGTTGGTTAGTTTTCCCTGTTTTAATTTTATTGGCTGGTGGTGGGATCGCTTTACCTGCATTACAGGGAGTGATGTCTATCCAAACAAAGAGTCATCAGCAAGGTGCTTTACAGGGATTATTGGTGAGCCTTACCAATGCAACCGGTGTTATTGGCCCATTACTGTTTGCTGTTATTTATAATCATTCACTACCAATTTGGGATGGCTGGATTTGGATTATTGGTTTAGCGTTTTACTGTATTATTATCCTGCTATCGATGACCTTCATGTTAACCCCTCAAGCTCAGGGGAGTAAACAGGAGACAAGTGCTTAG |

**Table 33:** Antibiotic resistance genes in 58E genome

| **Gene** | **Predicted Phenotype** | **CGE Predicted Phenotype** | **%Identity** | **Start** | **End** | **Accession** | **Sequence** |
| --- | --- | --- | --- | --- | --- | --- | --- |
| aph(3'')-Ib | streptomycin | Streptomycin | 99.88 | 2866 | 3669 | AF321551 | TTGAATCGAACTAATATTTTTTTGGGTGAATCGCATTCTGACTGGTTGCCTGTCAGAGGCGGAGAATCTGGTGATTTTGTTTTTCGACGTGGTGACGGGCATGCCTTCGCGAAAATCGCACCTGCTTCCCGCCGCGGTGAGCTCGCTGGAGAGCGTGACCGCCTCATTTGGCTCAAAGGTCGAGGTGTGGCTTGCCCCGAGGTCATCAACTGGCAGGAGGAACAGGAGGGTGCATGCTTGGTGATAACGGCAATTCCGGGAGTACCGGCGGCTGATCTGTCTGGAGCGGATTTGCTCAAAGCGTGGCCGTCAATGGGGCAGCAACTTGGCGCTGTTCACAGCCTATCGGTTGATCAATGTCCGTTTGAGCGCAGGCTGTCGCGAATGTTCGGACGCGCCGTTGATGTGGTGTCCCGCAATGCCGTCAATCCCGACTTCTTACCGGACGAGGACAAGAGTACGCCGCAGCTCGATCTTTTGGCTCGTGTCGAACGAGAGCTACCGGTGCGGCTCGACCAAGAGCGCACCGATATGGTTGTTTGCCATGGTGATCCCTGCATGCCGAACTTCATGGTGGACCCTAAAACTCTTCAATGCACGGGTCTGATCGACCTTGGGCGGCTCGGAACAGCAGATCGCTATGCCGATTTGGCACTCATGATTGCTAACGCCGAAGAGAACTGGGCAGCGCCAGATGAAGCAGAGCGCGCCTTCGCTGTCCTATTCAATGTATTGGGGATCGAAGCCCCCGACCGCGAACGCCTTGCCTTCTATCTGCGATTGGACCCTCTGACTTGGGGTTGA |
| aph(6)-Id | kanamycin | Streptomycin | 100 | 3669 | 4505 | M28829 | ATGTTCATGCCGCCTGTTTTTCCTGCTCATTGGCACGTTTCGCAACCTGTTCTCATTGCGGACACCTTTTCCAGCCTCGTTTGGAAAGTTTCATTGCCAGACGGGACTCCTGCAATCGTCAAGGGATTGAAACCTATAGAAGACATTGCTGATGAACTGCGCGGGGCCGACTATCTGGTATGGCGCAATGGGAGGGGAGCAGTCCGGTTGCTCGGTCGTGAGAACAATCTGATGTTGCTCGAATATGCCGGGGAGCGAATGCTCTCTCACATCGTTGCCGAGCACGGCGACTACCAGGCGACCGAAATTGCAGCGGAACTAATGGCGAAGCTGTATGCCGCATCTGAGGAACCCCTGCCTTCTGCCCTTCTCCCGATCCGGGATCGCTTTGCAGCTTTGTTTCAGCGGGCGCGCGATGATCAAAACGCAGGTTGTCAAACTGACTACGTCCACGCGGCGATTATAGCCGATCAAATGATGAGCAATGCCTCGGAACTGCGTGGGCTACATGGCGATCTGCATCATGAAAACATCATGTTCTCCAGTCGCGGCTGGCTGGTGATAGATCCCGTCGGTCTGGTCGGTGAAGTGGGCTTTGGCGCCGCCAATATGTTCTACGATCCGGCTGACAGAGACGACCTTTGTCTCGATCCTAGACGCATTGCACAGATGGCGGACGCATTCTCTCGTGCGCTGGACGTCGATCCGCGTCGCCTGCTCGACCAGGCGTACGCTTATGGGTGCCTTTCCGCAGCTTGGAACGCGGATGGAGAAGAGGAGCAACGCGATCTAGCTATCGCGGCCGCGATCAAGCAGGTGCGACAGACGTCATACTAG |
| qnrS1 | ciprofloxacin I/R | Ciprofloxacin | 100 | 9669 | 9013 | AB187515 | ATGGAAACCTACAATCATACATATCGGCACCACAACTTTTCACATAAAGACTTAAGTGATCTCACCTTCACCGCTTGCACATTCATTCGCAGCGACTTTCGACGTGCTAACTTGCGTGATACGACATTCGTCAACTGCAAGTTCATTGAACAGGGTGATATCGAAGGCTGCCACTTTGATGTCGCAGATCTTCGTGATGCAAGTTTCCAACAATGCCAACTTGCGATGGCAAACTTCAGTAATGCCAATTGCTACGGTATAGAGTTCCGTGCGTGTGATTTAAAAGGTGCCAACTTTTCCCGAACAAACTTTGCCCATCAAGTGAGTAATCGTATGTACTTTTGCTCAGCATTTATTTCTGGATGTAATCTTTCCTATGCCAATATGGAGAGGGTTTGTTTAGAAAAATGTGAGTTGTTTGAAAATCGCTGGATAGGAACGAACCTAGCGGGTGCATCACTGAAAGAGTCAGACTTAAGTCGAGGTGTTTTTTCCGAAGATGTCTGGGGGCAATTTAGCCTACAGGGTGCCAATTTATGCCACGCCGAACTCGACGGTTTAGATCCCCGCAAAGTCGATACATCAGGTATCAAAATTGCAGCCTGGCAGCAAGAACTGATTCTCGAAGCACTGGGTATTGTTGTTTATCCTGACTAA |
| sul2 | sulfisoxazole | Sulfamethoxazole | 100 | 1990 | 2805 | HQ840942 | ATGAATAAATCGCTCATCATTTTCGGCATCGTCAACATAACCTCGGACAGTTTCTCCGATGGAGGCCGGTATCTGGCGCCAGACGCAGCCATTGCGCAGGCGCGTAAGCTGATGGCCGAGGGGGCAGATGTGATCGACCTCGGTCCGGCATCCAGCAACCCCGACGCCGCGCCTGTTTCGTCCGACACAGAAATCGAGCGTATCGCGCCGGTGCTGGACGCGCTCAAGGCAGATGGCATTCCCGTCTCGCTCGACAGTTATCAACCCGCGACGCAAGCCTATGCCTTGTCGCGTGGTGTGGCCTATCTCAATGATATTCGCGGTTTTCCAGACGCTGCGTTCTATCCGCAATTGGCGAAATCATCTGCCAAACTCGTCGTTATGCATTCGGTGCAAGACGGGCAGGCAGATCGGCGCGAGGCACCCGCTGGCGACATCATGGATCACATTGCGGCGTTCTTTGACGCGCGCATCGCGGCGCTGACGGGTGCCGGTATCAAACGCAACCGCCTTGTCCTTGATCCCGGCATGGGGTTTTTTCTGGGGGCTGCTCCCGAAACCTCGCTCTCGGTGCTGGCGCGGTTCGATGAATTGCGGCTGCGCTTCGATTTGCCGGTGCTTCTGTCTGTTTCGCGCAAATCCTTTCTGCGCGCGCTCACAGGCCGTGGTCCGGGGGATGTCGGGGCCGCGACACTCGCTGCAGAGCTTGCCGCCGCCGCAGGTGGAGCTGACTTCATCCGCACACACGAGCCGCGCCCCTTGCGCGACGGGCTGGCGGTATTGGCGGCGCTAAAAGAAACCGCAAGAATTCGTTAA |
