# Supplementary figures and images for: Genomic and phenotypic characterization of plasmid-mediated extensively drug-resistant Salmonella Typhi from Lahore Pakistan carrying IncY IncQ1 and IncC replicons
Source: Sci Rep. 2026 Mar 16;16:13606. doi: 10.1038/s41598-026-37560-5 (PMC13121732; doi:10.1038/s41598-026-37560-5)

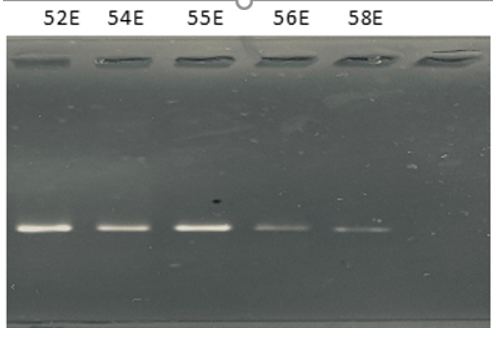

Supplement: Supplementary file 3 — Supplementary Material 3 [file 41598_2026_37560_MOESM3_ESM.png]

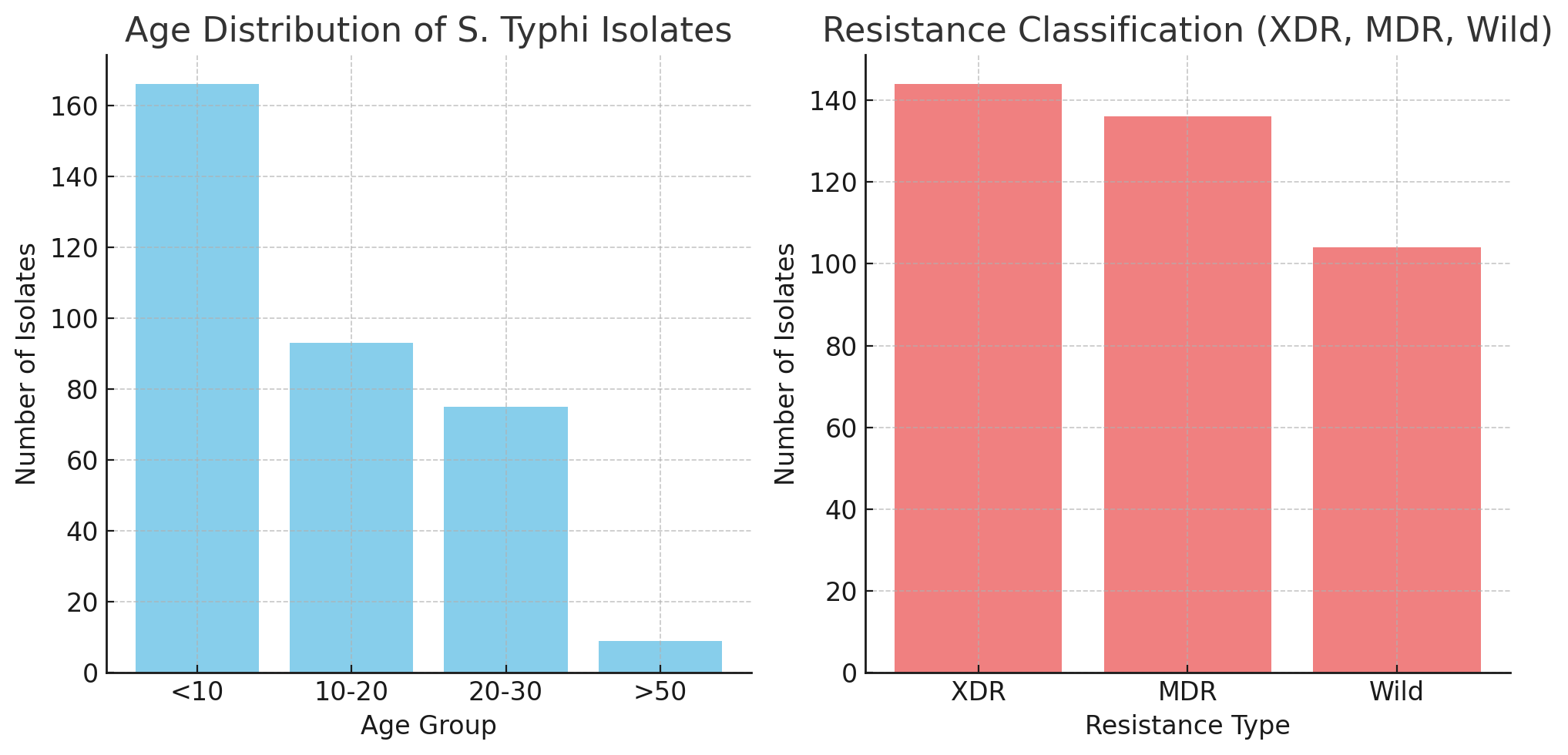

Supplement: Supplementary file 5 — Supplementary Material 5 [file 41598_2026_37560_MOESM5_ESM.png]
