# Supplementary material for: Genomic and phenotypic characterization of plasmid-mediated extensively drug-resistant Salmonella Typhi from Lahore Pakistan carrying IncY IncQ1 and IncC replicons
Source: Sci Rep. 2026 Mar 16;16:13606. doi: 10.1038/s41598-026-37560-5 (PMC13121732; doi:10.1038/s41598-026-37560-5)
Supplement: Supplementary file 4 — Supplementary Material 4 [file 41598_2026_37560_MOESM4_ESM.docx]

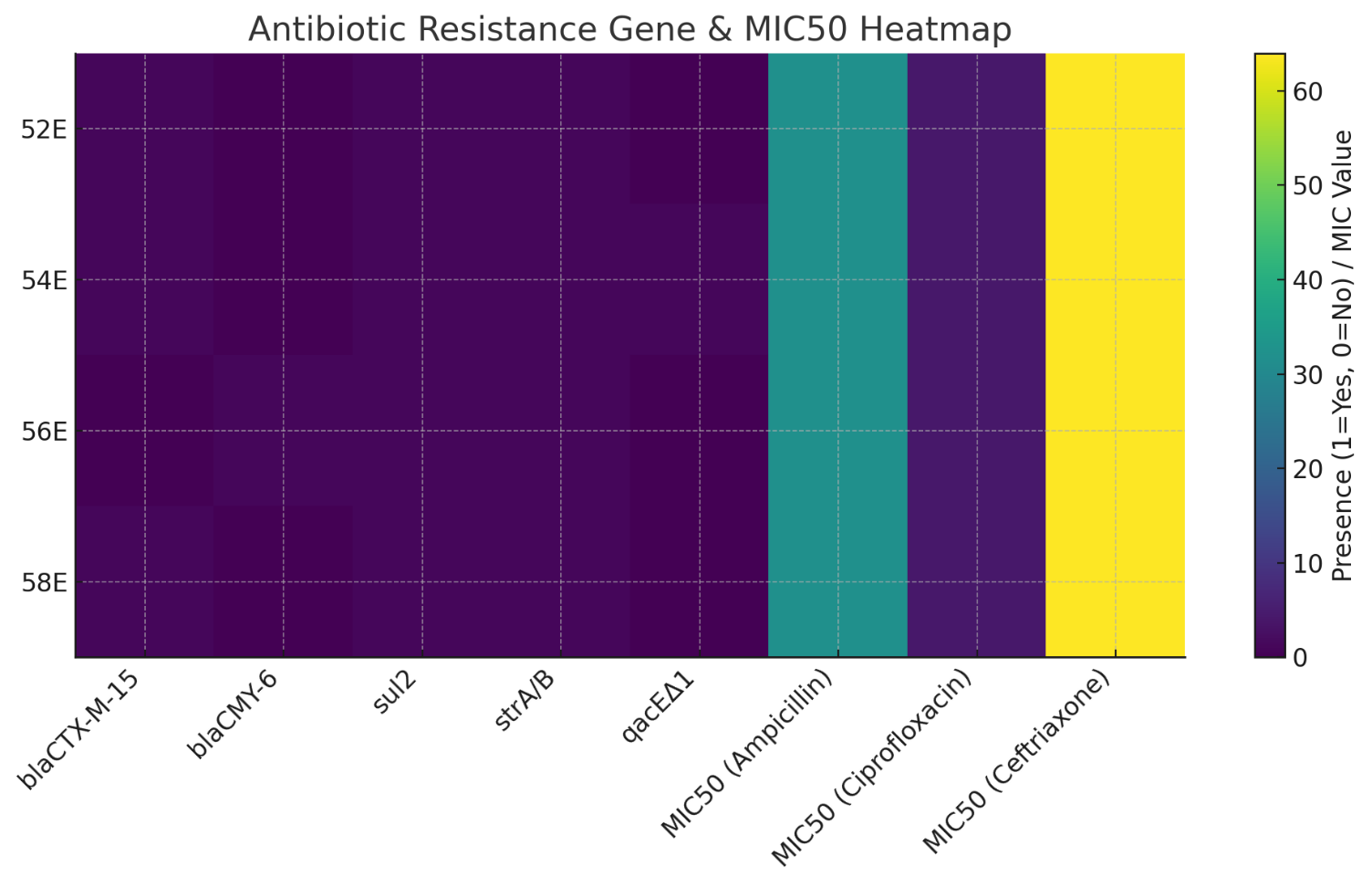


Heatmap showing the presence of resistance genes (Yes/No) and MIC50 values for each isolate. The lighter colors represent higher MIC values or presence of genes (Yes = 1), while darker colors show absence (No = 0)
